# Machine Learning in Agriculture: A Comprehensive Updated Review

**DOI:** 10.3390/s21113758

**Published:** 2021-05-28

**Authors:** Lefteris Benos, Aristotelis C. Tagarakis, Georgios Dolias, Remigio Berruto, Dimitrios Kateris, Dionysis Bochtis

**Affiliations:** 1 Centre of Research and Technology-Hellas (CERTH), Institute for Bio-Economy and Agri-Technology (IBO), 6th km Charilaou-Thermi Rd, GR 57001 Thessaloniki, Greece; e.benos@certh.gr (L.B.); a.tagarakis@certh.gr (A.C.T.); g.dolias@certh.gr (G.D.); d.kateris@certh.gr (D.K.); 2 Department of Agriculture, Forestry and Food Science (DISAFA), University of Turin, Largo Braccini 2, 10095 Grugliasco, Italy; remigio.berruto@unito.it; 3 FarmB Digital Agriculture P.C., Doiranis 17, GR 54639 Thessaloniki, Greece

**Keywords:** machine learning, crop management, water management, soil management, livestock management, artificial intelligence, precision agriculture, precision livestock farming

## Abstract

The digital transformation of agriculture has evolved various aspects of management into artificial intelligent systems for the sake of making value from the ever-increasing data originated from numerous sources. A subset of artificial intelligence, namely machine learning, has a considerable potential to handle numerous challenges in the establishment of knowledge-based farming systems. The present study aims at shedding light on machine learning in agriculture by thoroughly reviewing the recent scholarly literature based on keywords’ combinations of “machine learning” along with “crop management”, “water management”, “soil management”, and “livestock management”, and in accordance with PRISMA guidelines. Only journal papers were considered eligible that were published within 2018–2020. The results indicated that this topic pertains to different disciplines that favour convergence research at the international level. Furthermore, crop management was observed to be at the centre of attention. A plethora of machine learning algorithms were used, with those belonging to Artificial Neural Networks being more efficient. In addition, maize and wheat as well as cattle and sheep were the most investigated crops and animals, respectively. Finally, a variety of sensors, attached on satellites and unmanned ground and aerial vehicles, have been utilized as a means of getting reliable input data for the data analyses. It is anticipated that this study will constitute a beneficial guide to all stakeholders towards enhancing awareness of the potential advantages of using machine learning in agriculture and contributing to a more systematic research on this topic.

## 1. Introduction

### 1.1. General Context of Machine Learning in Agriculture

Modern agriculture has to cope with several challenges, including the increasing call for food, as a consequence of the global explosion of earth’s population, climate changes [1], natural resources depletion [2], alteration of dietary choices [3], as well as safety and health concerns [4]. As a means of addressing the above issues, placing pressure on the agricultural sector, there exists an urgent necessity for optimizing the effectiveness of agricultural practices by, simultaneously, lessening the environmental burden. In particular, these two essentials have driven the transformation of agriculture into precision agriculture. This modernization of farming has a great potential to assure sustainability, maximal productivity, and a safe environment [5]. In general, smart farming is based on four key pillars in order to deal with the increasing needs; (a) optimal natural resources’ management, (b) conservation of the ecosystem, (c) development of adequate services, and (d) utilization of modern technologies [6]. An essential prerequisite of modern agriculture is, definitely, the adoption of Information and Communication Technology (ICT), which is promoted by policy-makers around the world. ICT can indicatively include farm management information systems, humidity and soil sensors, accelerometers, wireless sensor networks, cameras, drones, low-cost satellites, online services, and automated guided vehicles [7].

The large volume of data, which is produced by digital technologies and usually referred to as “big data”, needs large storage capabilities in addition to editing, analyzing, and interpreting. The latter has a considerable potential to add value for society, environment, and decision-makers [8]. Nevertheless, big data encompass challenges on account of their so-called “5-V” requirements; (a) Volume, (b) Variety, (c) Velocity, (d) Veracity, and (e) Value [9]. The conventional data processing techniques are incapable of meeting the constantly growing demands in the new era of smart farming, which is an important obstacle for extracting valuable information from field data [10]. To that end, Machine Learning (ML) has emerged, which is a subset of artificial intelligence [11], by taking advantage of the exponential computational power capacity growth.

**Figure 1:**
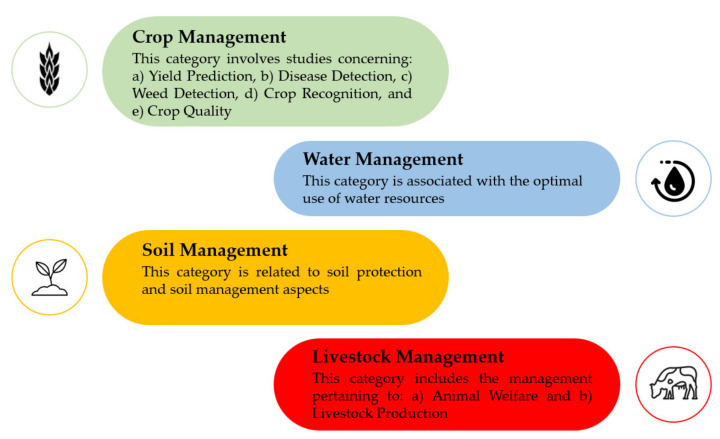
The four generic categories in agriculture exploiting machine learning techniques, as presented in [12].

There is a plethora of applications of ML in agriculture. According to the recent literature survey by Liakos et al. [12], regarding the time period of 2004 to 2018, four generic categories were identified (Figure 1). These categories refer to crop, water, soil, and livestock management. In particular, as far as crop management is concerned, it represented the majority of the articles amongst all categories (61% of the total articles) and was further sub-divided into:

- Yield prediction;
- Disease detection;
- Weed detection;
- Crop recognition;
- Crop quality.

The generic categories dealing with the management of water and soil were found to be less investigated, corresponding cumulatively to 20% of the total number of papers (10% for each category).

Finally, two main sub-categories were identified for the livestock-related applications corresponding to a total 19% of journal papers:

- Livestock production;
- Animal welfare.

### 1.2. Open Problems Associated with Machine Learning in Agriculture

Due to the broad range of applications of ML in agriculture, several reviews have been published in this research field. The majority of these review studies have been dedicated to crop disease detection [13,14,15,16], weed detection [17,18], yield prediction [19,20], crop recognition [21,22], water management [23,24], animal welfare [25,26], and livestock production [27,28]. Furthermore, other studies were concerned with the implementation of ML methods regarding the main grain crops by investigating different aspects including quality and disease detection [29]. Finally, focus has been paid on big data analysis using ML, aiming at finding out real-life problems that originated from smart farming [30], or dealing with methods to analyze hyperspectral and multispectral data [31].

Although ML in agriculture has made considerable progress, several open problems remain, which have some common points of reference, despite the fact that the topic covers a variety of sub-fields. According to [23,24,28,32], the main problems are associated with the implementation of sensors on farms for numerous reasons, including high costs of ICT, traditional practices, and lack of information. In addition, the majority of the available datasets do not reflect realistic cases, since they are normally generated by a few people getting images or specimens in a short time period and from a limited area [15,21,22,23]. Consequently, more practical datasets coming from fields are required [18,20]. Moreover, the need for more efficient ML algorithms and scalable computational architectures has been pointed out, which can lead to rapid information processing [18,22,23,31]. The challenging background, when it comes to obtaining images, video, or audio recordings, has also been mentioned owing to changes in lighting [16,29], blind spots of cameras, environmental noise, and simultaneous vocalizations [25]. Another important open problem is that the vast majority of farmers are non-experts in ML and, thus, they cannot fully comprehend the underlying patterns obtained by ML algorithms. For this reason, more user-friendly systems should be developed. In particular, simple systems, being easy to understand and operate, would be valuable, as for example a visualization tool with a user-friendly interface for the correct presentation and manipulation of data [25,30,31]. Taking into account that farmers are getting more and more familiar with smartphones, specific smartphone applications have been proposed as a possible solution to address the above challenge [15,16,21]. Last but not least, the development of efficient ML techniques by incorporating expert knowledge from different stakeholders should be fostered, particularly regarding computing science, agriculture, and the private sector, as a means of designing realistic solutions [19,22,24,33]. As stated in [12], currently, all of the efforts pertain to individual solutions, which are not always connected with the process of decision-making, as seen for example in other domains.

### 1.3. Aim of the Present Study

As pointed out above, because of the multiple applications of ML in agriculture, several review studies have been published recently. However, these studies usually concentrate purely on one sub-field of agricultural production. Motivated by the current tremendous progress in ML, the increasing interest worldwide, and its impact in various do-mains of agriculture, a systematic bibliographic survey is presented on the range of the categories proposed in [12], which were summarized in Figure 1. In particular, we focus on reviewing the relevant literature of the last three years (2018–2020) for the intention of providing an updated view of ML applications in agricultural systems. In fact, this work is an updated continuation of the work presented at [12]; following, consequently, exactly the same framework and inclusion criteria. As a consequence, the scholarly literature was screened in order to cover a broad spectrum of important features for capturing the current progress and trends, including the identification of: (a) the research areas which are interested mostly in ML in agriculture along with the geographical distribution of the contributing organizations, (b) the most efficient ML models, (c) the most investigated crops and animals, and (d) the most implemented features and technologies.

As will be discussed next, overall, a 745% increase in the number of journal papers took place in the last three years as compared to [12], thus justifying the need for a new updated review on the specific topic. Moreover, crop management remained as the most investigated topic, with a number of ML algorithms having been exploited as a means of tackling the heterogeneous data that originated from agricultural fields. As compared to [12], more crop and animal species have been investigated by using an extensive range of input parameters coming mainly from remote sensing, such as satellites and drones. In addition, people from different research fields have dealt with ML in agriculture, hence, contributing to the remarkable advancement in this field.

### 1.4. Outline of the Paper

The remainder of this paper is structured as follows. The second section briefly describes the fundamentals of ML along with the subject of the four generic categories for the sake of better comprehension of the scope of the present study. The implemented methodology, along with the inclusive criteria and the search engines, is analyzed in the third section. The main performance metrics, which were used in the selected articles, are also presented in this section. The main results are shown in the fourth section in the form of bar and pie charts, while in the fifth section, the main conclusions are drawn by also discussing the results from a broader perspective. Finally, all the selected journal papers are summarized in Table A1, Table A2, Table A3, Table A4, Table A5, Table A6, Table A7, Table A8 and Table A9, in accordance with their field of application, and presented in the Appendix A, together with Table A10 and Table A11 that contain commonly used abbreviations, with the intention of not disrupting the flow of the main text.

## 2. Background

### 2.1. Fundamentals of Machine Learning: A Brief Overview

In general, the objective of ML algorithms is to optimize the performance of a task, via exploiting examples or past experience. In particular, ML can generate efficient relationships regarding data inputs and reconstruct a knowledge scheme. In this data-driven methodology, the more data are used, the better ML works. This is similar to how well a human being performs a particular task by gaining more experience [34]. The central outcome of ML is a measure of generalizability; the degree to which the ML algorithm has the ability to provide correct predictions, when new data are presented, on the basis of learned rules originated from preceding exposure to similar data [35]. More specifically, data involve a set of examples, which are described by a group of characteristics, usually called features. Broadly speaking, ML systems operate at two processes, namely the learning (used for training) and testing. In order to facilitate the former process, these features commonly form a feature vector that can be binary, numeric, ordinal, or nominal [36]. This vector is utilized as an input within the learning phase. In brief, by relying on training data, within the learning phase, the machine learns to perform the task from experience. Once the learning performance reaches a satisfactory point (expressed through mathematical and statistical relationships), it ends. Subsequently, the model that was developed through the training process can be used to classify, cluster, or predict.

**Figure 2:**
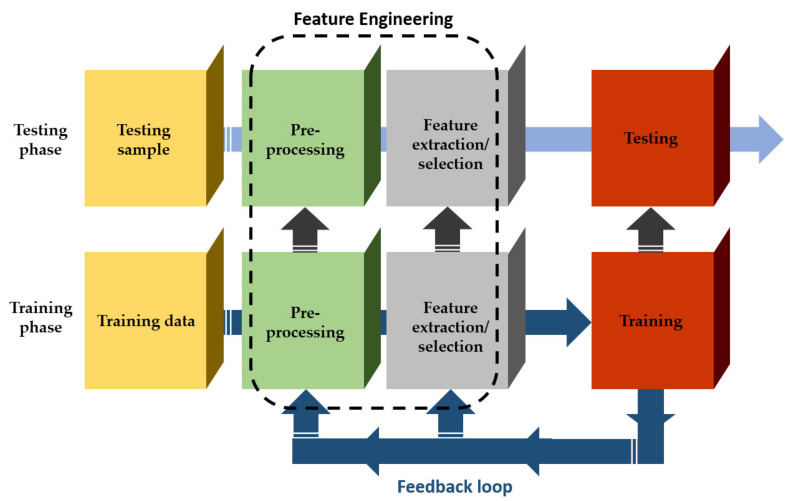
A graphical illustration of a typical machine learning system.

An overview of a typical ML system is illustrated in Figure 2. With the intention of forming the derived complex raw data into a suitable state, a pre-processing effort is required. This usually includes: (a) data cleaning for removing inconsistent or missing items and noise, (b) data integration, when many data sources exist and (c) data transformation, such as normalization and discretization [37]. The extraction/selection feature aims at creating or/and identifying the most informative subset of features in which, subsequently, the learning model is going to be implemented throughout the training phase [38]. Regarding the feedback loop, which is depicted in Figure 2, it serves for adjustments pertaining to the feature extraction/selection unit as well as the pre-processing one that further improves the overall learning model’s performance. During the phase of testing, previously unseen samples are imported to the trained model, which are usually represented as feature vectors. Finally, an appropriate decision is made by the model (for example, classification or regression) in reliance of the features existing in each sample. Deep learning, a subfield of ML, utilizes an alternative architecture via shifting the process of converting raw data to features (feature engineering) to the corresponding learning system. Consequently, the feature extraction/selection unit is absent, resulting in a fully trainable system; it starts from a raw input and ends with the desired output [39,40].

Based on the learning type, ML can be classified according to the relative literature [41,42] as:

- Supervised learning: The input and output are known and the machine tries to find the optimal way to reach an output given an input;
- Unsupervised learning: No labels are provided, leaving the learning algorithm itself to generate structure within its input;
- Semi-supervised learning: Input data constitute a mixture of labeled and non-labeled data;
- Reinforcement learning: Decisions are made towards finding out actions that can lead to the more positive outcome, while it is solely determined by trial and error method and delayed outcome.

Nowadays, ML is used in facilitating several management aspects in agriculture [12] and in a plethora of other applications, such as image recognition [43], speech recognition [44], autonomous driving [45], credit card fraud detection [46], stock market forecasting [47], fluid mechanics [48], email, spam and malware filtering [49], medical diagnosis [40], contamination detection in urban water networks [50], and activity recognition [51], to mention but a few.

### 2.2. Brief Description of the Four Generic Categories

#### 2.2.1. Crop Management

The crop management category involves versatile aspects that originated from the combination of farming techniques in the direction of managing the biological, chemical and physical crop environment with the aim of reaching both quantitative and qualitative targets [52]. Using advanced approaches to manage crops, such as yield prediction, disease detection, weed detection, crop recognition, and crop quality, contributes to the increase of productivity and, consequently, the financial income. The above aspects constitute key goals of precision agriculture.

##### Yield Prediction

In general, yield prediction is one of the most important and challenging topics in modern agriculture. An accurate model can help, for instance, the farm owners to take informed management decisions on what to grow towards matching the crop to the existing market’s demands [20]. However, this is not a trivial task; it consists of various steps. Yield prediction can be determined by several factors such as environment, management practices, crop genotypic and phenotypic characteristics, and their interactions. Hence, it necessitates a fundamental comprehension of the relationship between these interactive factors and yield. In turn, identifying such kinds of relationships mandates comprehensive datasets along with powerful algorithms such as ML techniques [53].

##### Disease Detection

Crop diseases constitute a major threat in agricultural production systems that deteriorate yield quality and quantity at production, storage, and transportation level. At farm level, reports on yield losses, due to plant diseases, are very common [54]. Furthermore, crop diseases pose significant risks to food security at a global scale. Timely identification of plant diseases is a key aspect for efficient management. Plant diseases may be provoked by various kinds of bacteria, fungi, pests, viruses, and other agents. Disease symptoms, namely the physical evidence of the presence of pathogens and the changes in the plants’ phenotype, may consist of leaf and fruit spots, wilting and color change [55], curling of leaves, etc. Historically, disease detection was conducted by expert agronomists, by performing field scouting. However, this process is time-consuming and solely based on visual inspection. Recent technological advances have made commercially available sensing systems able to identify diseased plants before the symptoms become visible. Furthermore, in the past few years, computer vision, especially by employing deep learning, has made remarkable progress. As highlighted by Zhang et al. [56], who focused on identifying cucumber leaf diseases by utilizing deep learning, due to the complex environmental background, it is beneficial to eliminate background before model training. Moreover, accurate image classifiers for disease diagnosis need a large dataset of both healthy and diseased plant images. In reference to large-scale cultivations, such kinds of automated processes can be combined with autonomous vehicles, to timely identify phytopathological problems by implementing regular inspections. Furthermore, maps of the spatial distribution of the plant disease can be created, depicting the zones in the farm where the infection has been spread [57].

##### Weed Detection

As a result of their prolific seed production and longevity, weeds usually grow and spread invasively over large parts of the field very fast, competing with crops for the resources, including space, sunlight, nutrients, and water availability. Besides, weeds frequently arise sooner than crops without having to face natural enemies, a fact that adversely affects crop growth [18]. In order to prevent crop yield reduction, weed control is an important management task by either mechanical treatment or application of herbicides. Mechanical treatment is, in most cases, difficult to be performed and ineffective if not properly performed, making herbicide application the most widely used operation. Using large quantities of herbicides, however, turns out to be both costly and detrimental for the environment, especially in the case of uniform application without taking into account the spatial distribution of the weeds. Remarkably, long-term herbicide use is very likely to make weeds more resistant, thus, resulting in more demanding and expensive weed control. In recent years, considerable achievements have been made pertaining to the differentiation of weeds from crops on the basis of smart agriculture. This discrimination can be accomplished by using remote or proximal sensing with sensors attached on satellites, aerial, and ground vehicles, as well as unmanned vehicles (both ground (UGV) and aerial (UAV)). The transformation of data gathered by UAVs into meaningful information is, however, still a challenging task, since both data collection and classification need painstaking effort [58]. ML algorithms coupled with imaging technologies or non-imaging spectroscopy can allow for real-time differentiation and localization of target weeds, enabling precise application of herbicides to specific zones, instead of spraying the entire fields [59] and planning of the shortest weeding path [60].

##### Crop Recognition

Automatic recognition of crops has gained considerable attention in several scientific fields, such as plant taxonomy, botanical gardens, and new species discovery. Plant species can be recognized and classified via analysis of various organs, including leaves, stems, fruits, flowers, roots, and seeds [61,62]. Using leaf-based plant recognition seems to be the most common approach by examining specific leaf’s characteristics like color, shape, and texture [63]. With the broader use of satellites and aerial vehicles as means of sensing crop properties, crop classification through remote sensing has become particularly popular. As in the above sub-categories, the advancement on computer software and image processing devices combined with ML has led to the automatic recognition and classification of crops.

##### Crop Quality

Crop quality is very consequential for the market and, in general, is related to soil and climate conditions, cultivation practices and crop characteristics, to name a few. High quality agricultural products are typically sold at better prices, hence, offering larger earnings to farmers. For instance, as regards fruit quality, flesh firmness, soluble solids content, and skin color are among the most ordinary maturity indices utilized for harvesting [64]. The timing of harvesting greatly affects the quality characteristics of the harvested products in both high value crops (tree crops, grapes, vegetables, herbs, etc.) and arable crops. Therefore, developing decision support systems can aid farmers in taking appropriate management decisions for increased quality of production. For example, selective harvesting is a management practice that may considerably increase quality. Furthermore, crop quality is closely linked with food waste, an additional challenge that modern agriculture has to cope with, since if the crop deviates from the desired shape, color, or size, it may be thrown away. Similarly to the above sub-section, ML algorithms combined with imaging technologies can provide encouraging results.

#### 2.2.2. Water Management

The agricultural sector constitutes the main consumer of available fresh water on a global scale, as plant growth largely relies on water availability. Taking into account the rapid depletion rate of a lot of aquifers with negligible recharge, more effective water management is needed for the purpose of better conserving water in terms of accomplishing a sustainable crop production [65]. Effective water management can also lead to the improvement of water quality as well as reduction of pollution and health risks [66]. Recent research on precision agriculture offers the potential of variable rate irrigation so as to attain water savings. This can be realized by implementing irrigation at rates, which vary according to field variability on the basis of specific water requirements of separate management zones, instead of using a uniform rate in the entire field. The effectiveness and feasibility of the variable rate irrigation approach depend on agronomic factors, including topography, soil properties, and their effect on soil water in order to accomplish both water savings and yield optimization [67]. Carefully monitoring the status of soil water, crop growth conditions, and temporal and spatial patterns in combination with weather conditions monitoring and forecasting, can help in irrigation programming and efficient management of water. Among the utilized ICTs, remote sensing can provide images with spatial and temporal variability associated with the soil moisture status and crop growth parameters for precision water management. Interestingly, water management is challenging enough in arid areas, where groundwater sources are used for irrigation, with the precipitation providing only part of the total crop evapotranspiration (ET) demands [68].

#### 2.2.3. Soil Management

Soil, a heterogeneous natural resource, involves mechanisms and processes that are very complex. Precise information regarding soil on a regional scale is vital, as it contributes towards better soil management consistent with land potential and, in general, sustainable agriculture [5]. Better management of soil is also of great interest owing to issues like land degradation (loss of the biological productivity), soil-nutrient imbalance (due to fertilizers overuse), and soil erosion (as a result of vegetation overcutting, improper crop rotations rather than balanced ones, livestock overgrazing, and unsustainable fallow periods) [69]. Useful soil properties can entail texture, organic matter, and nutrients content, to mention but a few. Traditional soil assessment methods include soil sampling and laboratory analysis, which are normally expensive and take considerable time and effort. However, remote sensing and soil mapping sensors can provide low-cost and effortless solution for the study of soil spatial variability. Data fusion and handling of such heterogeneous “big data” may be important drawbacks, when traditional data analysis methods are used. ML techniques can serve as a trustworthy, low-cost solution for such a task.

#### 2.2.4. Livestock Management

It is widely accepted that livestock production systems have been intensified in the context of productivity per animal. This intensification involves social concerns that can influence consumer perception of food safety, security, and sustainability, based on animal welfare and human health. In particular, monitoring both the welfare of animals and overall production is a key aspect so as to improve production systems [70]. The above fields take place in the framework of precision livestock farming, aiming at applying engineering techniques to monitor animal health in real time and recognizing warning messages, as well as improving the production at the initial stages. The role of precision livestock farming is getting more and more significant by supporting the decision-making processes of livestock owners and changing their role. It can also facilitate the products’ traceability, in addition to monitoring their quality and the living conditions of animals, as required by policy-makers [71]. Precision livestock farming relies on non-invasive sensors, such as cameras, accelerometers, gyroscopes, radio-frequency identification systems, pedometers, and optical and temperature sensors [25]. IoT sensors leverage variable physical quantities (VPQs) as a means of sensing temperature, sound, humidity, etc. For instance, IoT sensors can warn if a VPQ falls out of regular limits in real-time, giving valuable information regarding individual animals. As a result, the cost of repetitively and arduously checking each animal can be reduced [72]. In order to take advantage of the large amounts of data, ML methodologies have become an integral part of modern livestock farming. Models can be developed that have the capability of defining the manner a biological system operates, relying on causal relationships and exploiting this biological awareness towards generating predictions and suggestions.

##### Animal Welfare

There is an ongoing concern for animal welfare, since the health of animals is strongly associated with product quality and, as a consequence, predominantly with the health of consumers and, secondarily, with the improvement of economic efficiency [73]. There exist several indexes for animal welfare evaluation, including physiological stress and behavioral indicators. The most commonly used indicator is animal behavior, which can be affected by diseases, emotions, and living conditions, which have the potential to demonstrate physiological conditions [25]. Sensors, commonly used to detect behavioral changes (for example, changes in water or food consumption, reduced animal activity), include microphone systems, cameras, accelerometers, etc.

##### Livestock Production

The use of sensor technology, along with advanced ML techniques, can increase livestock production efficiency. Given the impact of practices of animal management on productive elements, livestock owners are getting cautious of their asset. However, as the livestock holdings get larger, the proper consideration of every single animal is very difficult. From this perspective, the support to farmers via precision livestock farming, mentioned above, is an auspicious step for aspects associated with economic efficiency and establishment of sustainable workplaces with reduced environmental footprint [74]. Generally, several models have been used in animal production, with their intentions normally revolving around growing and feeding animals in the best way. However, the large volumes of data being involved, again, call for ML approaches.

## 3. Methods

### 3.1. Screening of the Relative Literature

**Figure 3:**
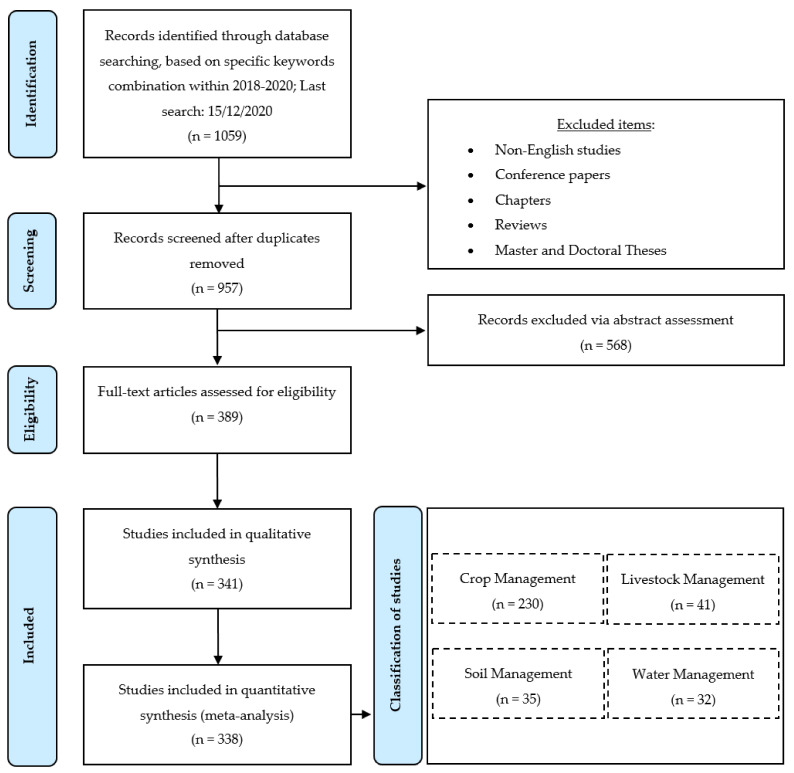
The flowchart of the methodology of the present systematic review along with the flow of information regarding the exclusive criteria, based on PRISMA guidelines [75].

In order to identify the relevant studies concerning ML in respect to different aspects of management in agriculture, the search engines of Scopus, Google Scholar, ScienceDirect, PubMed, Web of Science, and MDPI were utilized. In addition, keywords’ combinations of “machine learning” in conjunction with each of the following: “crop management”, “water management”, “soil management”, and “livestock management” were used. Our intention was to filter the literature on the same framework as [12]; however, focusing solely within the period 2018–2020. Once a relevant study was being identified, the references of the paper at hand were being scanned to find studies that had not been found throughout the initial searching procedure. This process was being iterated until no relevant studies occurred. In this stage, only journal papers were considered eligible. Thus, non-English studies, conferences papers, chapters, reviews, as well as Master and Doctoral Theses were excluded. The latest search was conducted on 15 December 2020. Subsequently, the abstract of each paper was being reviewed, while, at a next stage, the full text was being read to decide its appropriateness. After a discussion between all co-authors with reference to the appropriateness of the selected papers, some of them were excluded, in the case they did not meet the two main inclusion criteria, namely: (a) the paper was published within 2018–2020 and (b) the paper referred to one of the categories and sub-categories, which were summarized in Figure 1. Finally, the papers were classified in these sub-categories. Overall, 338 journal papers were identified. The flowchart of the present review methodology is depicted in Figure 3, based on the PRISMA guidelines [75], along with information about at which stage each exclusive criterion was imposed similarly to recent systematic review studies such as [72,76,77,78].

### 3.2. Definition of the Performance Metrics Commonly Used in the Reviewed Studies

In this subsection, the most commonly used performance metrics of the reviewed papers are briefly described. In general, these metrics are utilized in an effort to provide a common measure to evaluate the ML algorithms. The selection of the appropriate metrics is very important, since: (a) how the algorithm’s performance is measured relies on these metrics and (b) the metric itself can influence the way the significance of several characteristics is weighted.

**Figure 4:**
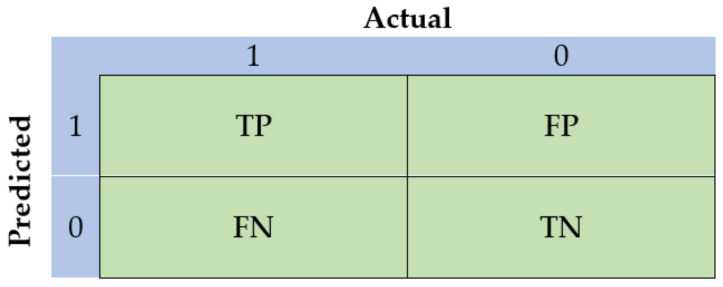
Representative illustration of a simplified confusion matrix.

Confusion matrix constitutes one of the most intuitive metrics towards finding the correctness of a model. It is used for classification problems, where the result can be of at least two types of classes. Let us consider a simple example, by giving a label to a target variable: for example, “1” when a plant has been infected with a disease and “0” otherwise. In this simplified case, the confusion matrix (Figure 4) is a 2 × 2 table having two dimensions, namely “Actual” and “Predicted”, while its dimensions have the outcome of the comparison between the predictions with the actual class label. Concerning the above simplified example, this outcome can acquire the following values:

- True Positive (TP): The plant has a disease (1) and the model classifies this case as diseased (1);
- True Negative (TN): The plant does not have a disease (0) and the model classifies this case as a healthy plant (0);
- False Positive (FP): The plant does not have a disease (0), but the model classifies this case as diseased (1);
- False Negative (FN): The plant has a disease (1), but the model classifies this case as a healthy plant (0).

As can be shown in Table 1, the aforementioned values can be implemented in order to estimate the performance metrics, typically observed in classification problems [79].

Other common evaluation metrics were the coefficient of correlation (R), coefficient of determination ($R^{2}$; basically, the square of the correlation coefficient), Mean Absolute Error (MAE), Mean Absolute Percentage Error (MAPE), and Mean Squared Error (MSE), which can be given via the following relationships [80,81]:

(1) $$R=\frac{T\cdot \sum_{t=1}^{T}Z\left(t\right)\cdot X\left(t\right)-\left(\sum_{t=1}^{T}Z\left(t\right)\right)\cdot \left(\sum_{t=1}^{T}X\left(t\right)\right)}{\sqrt{T\cdot \sum_{t=1}^{T}{\left(Z\left(t\right)\right)}^{2}-{\left(\sum_{t=1}^{T}Z\left(t\right)\right)}^{2}}\cdot \sqrt{T\cdot \sum_{t=1}^{T}{\left(X\left(t\right)\right)}^{2}-{\left(\sum_{t=1}^{T}X\left(t\right)\right)}^{2}}},$$

(2) $$MAE=\frac{1}{T}\cdot \sum_{t=1}^{T}\left|Z\left(t\right)-X\left(t\right)\right|,$$

(3) $$MAPE=\frac{1}{T}\cdot \sum_{t=1}^{T}\left|\frac{Z\left(t\right)-X\left(t\right)}{Z\left(t\right)}\right|,$$

(4) $$MSE=\frac{1}{T}\cdot \sum_{t=1}^{T}{\left(Z\left(t\right)-X\left(t\right)\right)}^{2},$$

where $X\left(t\right)$ and $Z\left(t\right)$ correspond to the predicted and real value, respectively, t stands for the iteration at each point, while T for the testing records number. Accordingly, low values of MAE, MAPE, and MSE values denote a small error and, hence, better performance. In contrast, $R^{2}$ near 1 is desired, which demonstrates better model performance and also that the regression curve efficiently fits the data.

## 4. Results

### 4.1. Preliminary Data Visualization Analysis

Graphical representation of data related to the reviewed studies, by using maps, bar or pie charts, for example, can provide an efficient approach to demonstrate and interpret the patterns of data. The data visualization analysis, as it usually refers to, can be vital in the context of analyzing large amounts of data and has gained remarkable attention in the past few years, including review studies. Indicatively, significant results can be deduced in an effort to identify: (a) the most contributing authors and organizations, (b) the most contributing international journals (or equivalently which research fields are interested in this topic), and (c) the current trends in this field [82].

#### 4.1.1. Classification of the Studies in Terms of Application Domain

**Figure 5:**
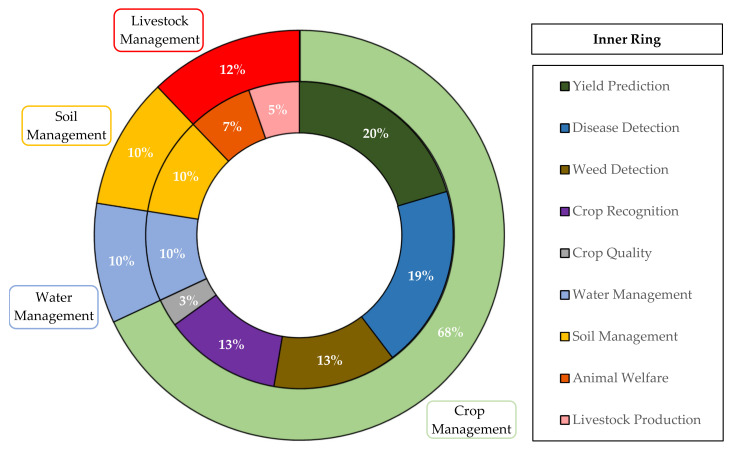
The classification of the reviewed studies according to the field of application.

As can be seen in the flowchart of the present methodology (Figure 3), the literature survey on ML in agriculture resulted in 338 journal papers. Subsequently, these studies were classified into the four generic categories as well as into their sub-categories, as already mentioned above. Figure 5 depicts the aforementioned papers’ distribution. In particular, the majority of the studies were intended for crop management (68%), while soil management (10%), water management (10%), and livestock management (12% in total; animal welfare: 7% and livestock production: 5%) had almost equal contribution in the present bibliographic survey. Focusing on crop management, the most contributing sub-categories were yield prediction (20%) and disease detection (19%). The former research field arises as a consequence of the increasing interest of farmers in taking decisions based on efficient management that can lead to the desired yield. Disease detection, on the other hand, is also very important, as diseases constitute a primary menace for food security and quality assurance. Equal percentages (13%) were observed for weed detection and crop recognition, both of which are essential in crop management at farm and agricultural policy making level. Finally, examination of crop quality was relatively scarce corresponding to 3% of all studies. This can be attributed to the complexity of monitoring and modeling the quality-related parameters.

In this fashion, it should be mentioned again that all the selected journal papers are summarized in Table A1, Table A2, Table A3, Table A4, Table A5, Table A6, Table A7, Table A8 and Table A9, depending on their field of application, and presented in the Appendix A. The columns of the tables correspond (from left to right) to the “Reference number” (Ref), “Input Data”, “Functionality”, “Models/Algorithms”, and “Best Output”. One additional column exists for the sub-categories belonging in crop management, namely “Crop”, whereas the corresponding column in the sub-categories pertaining to livestock management refers to “Animal”. The present systematic review deals with a plethora of different ML models and algorithms. For the sake of brevity, the commonly used abbreviations are used instead of the entire names, which are summarized in Table A10 and Table A11 (presented also in the Appendix A). The list of the aforementioned Tables, along with their content, is listed in Table 2.

#### 4.1.2. Geographical Distribution of the Contributing Organizations

**Figure 6:**
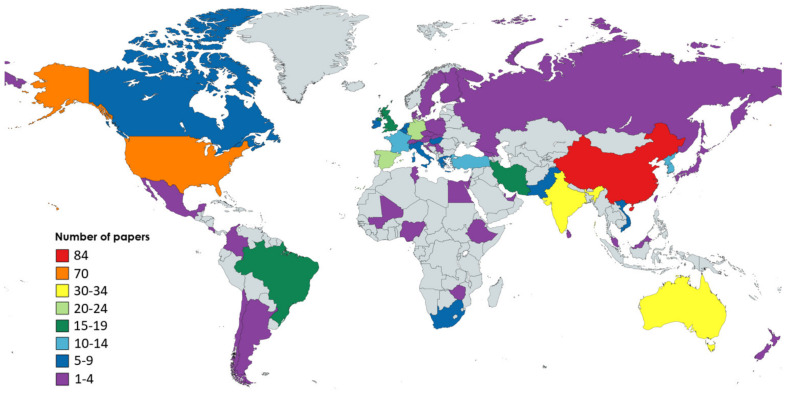
Geographical distribution of the contribution of each country to the research field focusing on machine learning in agriculture.

The subject of this sub-section is to find out the geographical distribution of all the contributing organizations in ML applications in agriculture. To that end, the author’s affiliation was taken into account. In case a paper included more than one author, which was the most frequent scenario, each country could contribute only once in the final map chart (Figure 6), similarly to [83,84]. As can be gleaned from Figure 6, investigating ML in agriculture is distributed worldwide, including both developed and developing economies. Remarkably, out of the 55 contributing countries, the least contribution originated from African countries (3%), whereas the major contribution came from Asian countries (55%). The latter result is attributed mainly to the considerable contribution of Chinese (24.9%) as well as Indian organizations (10.1%). USA appeared to be the second most contributing country with 20.7% percentage, while Australia (9.5%), Spain (6.8%), Germany (5.9%), Brazil, UK, and Iran (5.62%) seem to be particularly interested in ML in agriculture. It should be stressed that livestock management, which is a relatively different sub-field comparing to crop, water, and soil management, was primary examined from studies coming from Australia, USA, China, and UK, while all the papers regarding Ireland were focused on animals. Finally, another noteworthy observation is that a large number of articles were a result of international collaboration, with the synergy of China and USA standing out.

#### 4.1.3. Distribution of the Most Contributing Journal Papers

**Figure 7:**
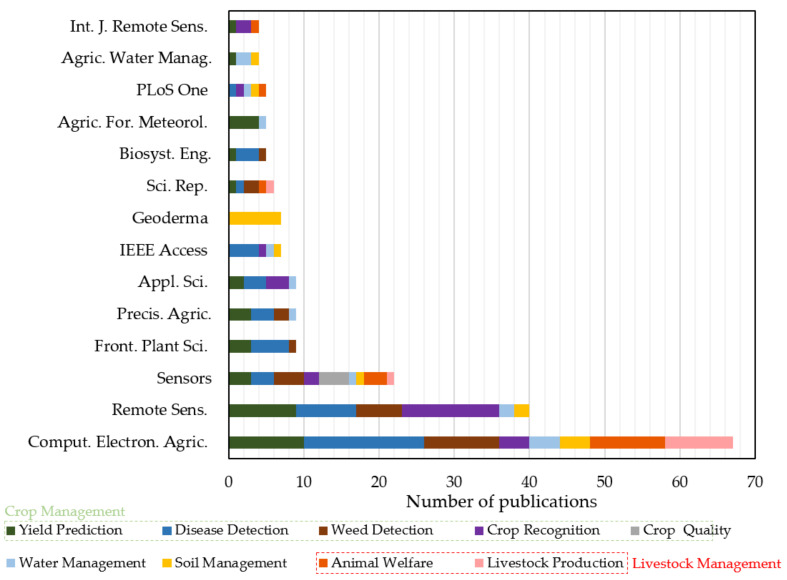
Distribution of the most contributing international journals (published at least four articles) concerning applications of machine learning in agriculture.

For the purpose of identifying the research areas that are mostly interested in ML in agriculture, the most frequently appeared international journal papers are depicted in Figure 7. In total, there were 129 relevant journals. However, in this bar chart, only the journals contributing with at least 4 papers are presented for brevity. As a general remark, remote sensing was of particular importance, since reliable data from satellites and UAV, for instance, constitute valuable input data for the ML algorithms. In addition, smart farming, environment, and agricultural sustainability were of central interest. Journals associated with computational techniques were also presented with considerable frequency. A typical example of such type of journals, which was presented in the majority of the studies with a percentage of 19.8%, was “*Computers and Electronics in Agriculture*”. This journal aims at providing the advances in relation to the application of computers and electronic systems for solving problems in plant and animal production.

The “*Remote Sensing*” and “*Sensors*” journals followed with approximately 11.8% and 6.5% of the total number of publications, respectively. These are cross-sectoral journals that are concentrated on applications of science and sensing technologies in various fields, including agriculture. Other journals, covering this research field, were also “*IEEE Access*” and “*International Journal of Remote Sensing*” with approximately 2.1% and 1.2% contribution, respectively. Moreover, agriculture-oriented journals were also presented in Figure 7, including “*Precision Agriculture*”, “*Frontiers in Plant Science*”, “*Agricultural and Forest Meteorology*”, and “*Agricultural Water Management*” with 1–3% percentage. These journals deal with several aspects of agriculture ranging from management strategies (so as to incorporate spatial and temporal data as a means of optimizing productivity, resource use efficiency, sustainability and profitability of agricultural production) up to crop molecular genetics and plant pathogens. An interdisciplinary journal concentrating on soil functions and processes also appeared with 2.1%, namely “*Geoderma*”, plausibly covering the soil management generic category. Finally, several journals focusing on physics and applied natural sciences, such as “*Applied Sciences*” (2.7%), “*Scientific Reports*” (1.8%), “*Biosystems Engineering*” (1.5%), and “*PLOS ONE*” (1.5%), had a notable contribution to ML studies. As a consequence, ML in agriculture concerns several disciplines and constitutes a fundamental area for developing various techniques, which can be beneficial to other fields as well.

### 4.2. Synopsis of the Main Features Associated with the Relative Literature

#### 4.2.1. Machine Learning Models Providing the Best Results

**Figure 8:**
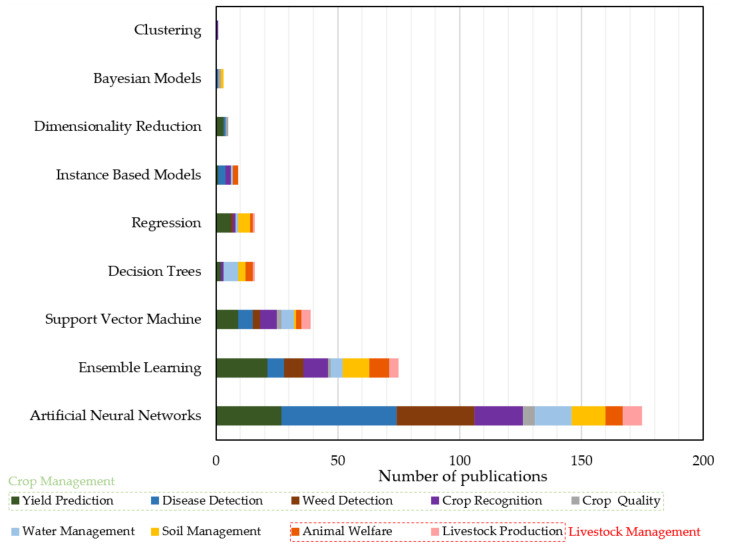
Machine Learning models giving the best output.

A wide range of ML algorithms was implemented in the selected studies; their abbreviations are given in Table A11. The ML algorithms that were used by each study as well as those that provided the best output have been listed in the last two columns of Table A1, Table A2, Table A3, Table A4, Table A5, Table A6, Table A7, Table A8 and Table A9. These algorithms can be classified into the eight broad families of ML models, which are summarized in Table A10. Figure 8 focuses on the best performed ML models as a means of capturing a broad picture of the current situation and demonstrating advancement similarly to [12].

As can be demonstrated in Figure 8, the most frequent ML model providing the best output was, by far, Artificial Neural Networks (ANNs), which appeared in almost half of the reviewed studies (namely, 51.8%). More specifically, ANN models provided the best results in the majority of the studies concerning all sub-categories. ANNs have been inspired by the biological neural networks that comprise human brains [85], while they allow for learning via examples from representative data describing a physical phenomenon. A distinct characteristic of ANNs is that they can develop relationships between dependent and independent variables, and thus extract useful information from representative datasets. ANN models can offer several benefits, such as their ability to handle noisy data [86], a situation that is very common in agricultural measurements. Among the most popular ANNs are the Deep Neural Networks (DNNs), which utilize multiple hidden layers between input and output layers. DNNs can be unsupervised, semi-supervised, or supervised. A usual kind of DNNs are the Convolutional Neural Networks (CNNs), whose layers, unlike common neural networks, can set up neurons in three dimensions [87]. In fact, CNNs were presented as the algorithms that provide the best output in all sub-categories, with an almost 50% of the individual percentage of ANNs. As stressed in recent studies, such as that of Yang et al. [88], CNNs are receiving more and more attention because of their efficient results when it comes to detection through images’ processing.

Recurrent Neural Networks (RNNs) followed, representing approximately 10% of ANNs, with Long Short-Term Memory (LSTM) standing out. They are called “recurrent” as they carry out the same process for every element, with the previous computations determining the current output, while they have a “memory” that stores information pertaining to what has been calculated so far. RNNs can face problems concerning vanishing gradients and inability to “memorize” many sequential data. Towards addressing these issues, the cell structures of LSTM can control which part of information will be either stored in long memory or discarded, resulting in optimization of the memorizing process [51]. Moreover, Multi-Layer Perceptron (MLP), Fully Convolutional Networks (FCNs), and Radial Basis Function Networks (RBFNs) appeared to have the best performance in almost 3–5% of ANNs. Finally, ML algorithms, belonging to ANNs with low frequency, were Back-Propagation Neural Networks (BPNNs), Modular Artificial Neural Networks (MANNs), Deep Belief Networks (DBNs), Adaptive-Neuro Fuzzy Inference System (ANFIS), Subtractive Clustering Fuzzy Inference System (SCFIS), Takagi-Sugeno Fuzzy Neural Networks (TS-FNN), and Feed Forward Neural Networks (FFNNs).

The second most accurate ML model was Ensemble Learning (EL), contributing to the ML models used in agricultural systems with approximately 22.2%. EL is a concise term for methods that integrate multiple inducers for the purpose of making a decision, normally in supervised ML tasks. An inducer is an algorithm, which gets as an input a number of labeled examples and creates a model that can generalize these examples. Thus, predictions can be made for a set of new unlabeled examples. The key feature of EL is that via combining various models, the errors coming from a single inducer is likely to be compensated from other inducers. Accordingly, the prediction of the overall performance would be superior comparing to a single inducer [89]. This type of ML model was presented in all sub-categories, apart from crop quality, perhaps owing to the small number of papers belonging in this subcategory. Support Vector Machine (SVM) followed, contributing in approximately 11.5% of the studies. The strength of the SVM stems from its capability to accurately learn data patterns while showing reproducibility. Despite the fact that it can also be applied for regression applications, SVM is a commonly used methodology for classification extending across numerous data science settings [90], including agricultural research.

Decision Trees (DT) and Regression models came next with equal percentage, namely 4.7%. Both these ML models were presented in all generic categories. As far as DT are concerned, they are either regression or classification models structured in a tree-like architecture. Interestingly, handling missing data in DT is a well-established problem. By implementing DT, the dataset can be gradually organized into smaller subsets, whereas, in parallel, a tree graph is created. In particular, each tree’s node denotes a dissimilar pairwise comparison regarding a certain feature, while each branch corresponds to the result of this comparison. As regards leaf nodes, they stand for the final decision/prediction provided after following a certain rule [91,92]. As for Regression, it is used for supervised learning models intending to model a target value on the basis of independent predictors. In particular, the output can be any number based on what it predicts. Regression is typically applied for time series modeling, prediction, and defining the relationships between the variables.

Finally, the ML models, leading to optimal performance (although with lower contribution to literature), were those of Instance Based Models (IBM) (2.7%), Dimensionality Reduction (DR) (1.5%), Bayesian Models (BM) (0.9%), and Clustering (0.3%). IBM appeared only in crop, water, and livestock management, whereas BM only in crop and soil management. On the other hand, DR and Clustering appeared as the best solution only in crop management. In brief, IBM are memory-based ML models that can learn through comparison of the new instances with examples within the training database. DR can be executed both in unsupervised and supervised learning types, while it is typically carried out in advance of classification/regression so as to prevent dimensionality effects. Concerning the case of BM, they are a family of probabilistic models whose analysis is performed within the Bayesian inference framework. BM can be implemented in both classification and regression problems and belong to the broad category of supervised learning. Finally, Clustering belongs to unsupervised ML models. It contains automatically discovering of natural grouping of data [12].

#### 4.2.2. Most Studied Crops and Animals

**Figure 9:**
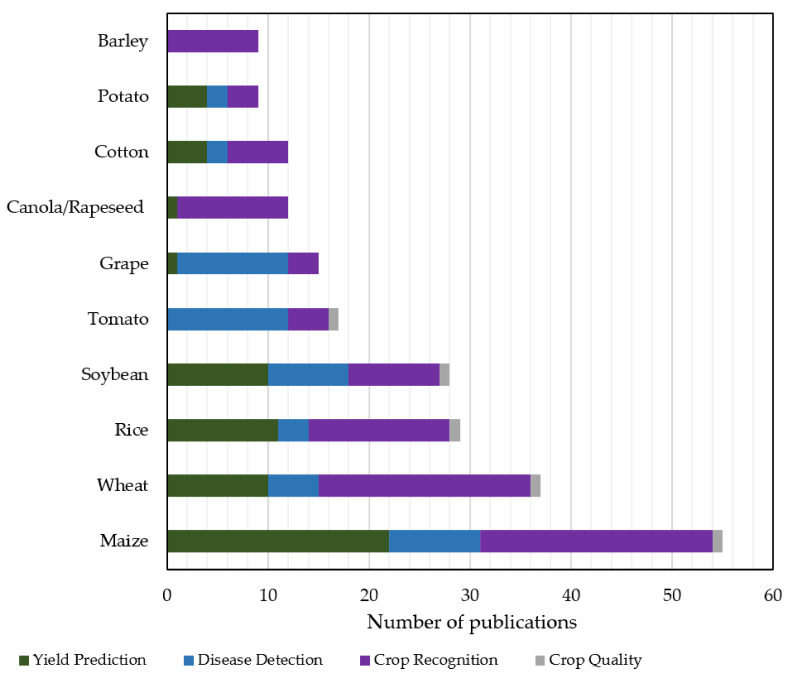
The 10 most investigated crops using machine learning models; the results refer to crop management.

In this sub-section, the most examined crops and animals that were used in the ML models are discussed as a result of our searching within the four sub-categories of crop management similarly to [12]. These sub-categories refer to yield prediction, disease detection, crop recognition, and crop quality. Overall, approximately 80 different crop species were investigated. The 10 most utilized crops are summarized in Figure 9. Specifically, the remarkable interest on maize (also known as corn) can be attributed to the fact that it is cultivated in many parts across the globe as well as its versatile usage (for example, direct consumption by humans, animal feed, producing ethanol, and other biofuels). Wheat and rice follow, which are two of the most widely consumed cereal grains. According to the Food and Agriculture Organization (FAO) [93], the trade in wheat worldwide is more than the summation of all other crops. Concerning rice, it is the cereal grain with the third-highest production and constitutes the most consumed staple food in Asia [94]. The large contribution of Asian countries presented in Figure 6, like China and India, justifies the interest in this crop. In the same vein, soybeans, which are broadly distributed in East Asia, USA, Africa, and Australia [95], were presented in many studies. Finally, tomato, grape, canola/rapeseed (cultivated primarily for its oil-rich seed), potato, cotton, and barley complete the top 10 examined crops. All these species are widely cultivated all over the world. Some other indicative species, which were investigated at least five times in the present reviewed studies, were also alfalfa, citrus, sunflower, pepper, pea, apple, squash, sugarcane, and rye.

**Figure 10:**
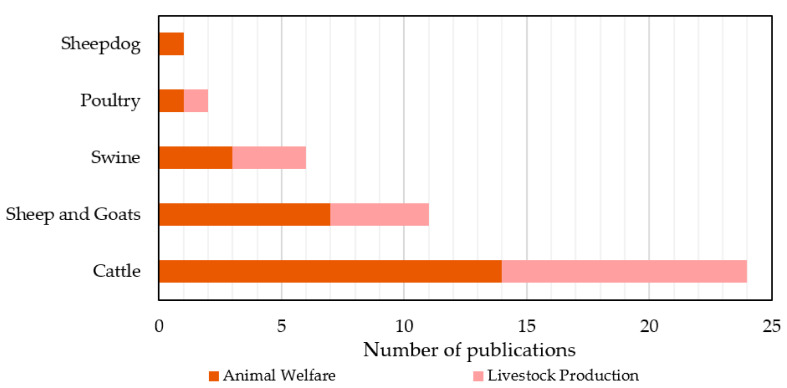
Frequency of animal species in studies concerning livestock management by using machine learning models.

As far as livestock management is concerned, the examined animal species can be classified, in descending order of frequency, into the categories of cattle (58.5%), sheep and goats (26.8%), swine (14.6%), poultry (4.9%), and sheepdog (2.4%). As can be depicted in Figure 10, the last animal, which is historically utilized with regard to the raising of sheep, was investigated only in one study belonging to animal welfare, whereas all the other animals were examined in both categories of livestock management. In particular, the most investigated animal in both animal welfare and livestock production was cattle. Sheep and goats came next, which included nine studies for sheep and two studies for goats. Cattles are usually raised as livestock aimed at meat, milk, and hide used for leather. Similarly, sheep are raised for meat and milk as well as fleece. Finally, swine (often called domestic pigs) and poultry (for example, chicken, turkey, and duck), which are used mainly for their meat or eggs (poultry), had equal contribution from the two livestock sub-categories.

#### 4.2.3. Most Studied Features and Technologies

**Figure 11:**
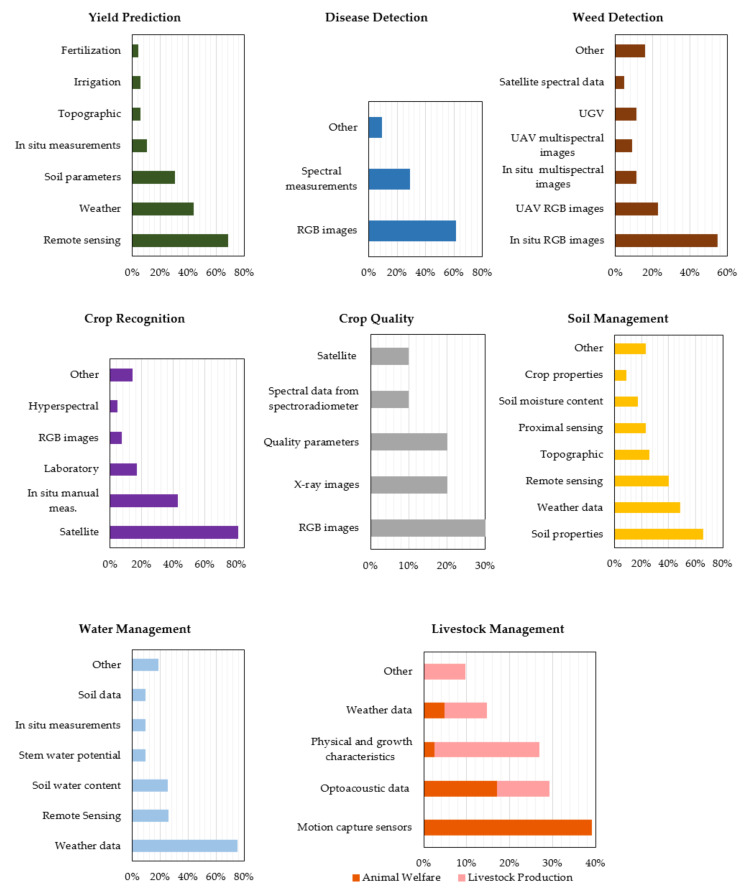
Distribution of the most usual features implemented as input data in the machine learning algorithms for each category/sub-category.

As mentioned in the beginning of this study, modern agriculture has to incorporate large amounts of heterogeneous data, which have originated from a variety of sensors over large areas at various spatial scale and resolution. Subsequently, such data are used as input into ML algorithms for their iterative learning up until modeling of the process in the most effective way possible. Figure 11 shows the features and technologies that were used in the reviewed studies, separately for each category, for the sake of better comprehending the results of the analysis.

Data coming from remote sensing were the most common in the yield prediction sub-category. Remote sensing, in turn, was primarily based on data derived from satellites (40.6% of the total studies published in this sub-category) and, secondarily, from UAVs (23.2% of the total studies published in this sub-category). A remarkable observation is the rapid increase of the usage of UAVs versus satellites from the year 2018 towards 2020, as UAVs seem to be a reliable alternative that can give faster and cheaper results, usually in higher resolution and independent of the weather conditions. Therefore, UAVs allow for discriminating details of localized circumscribed regions that the satellites’ lowest resolution may miss, especially under cloudy conditions. This explosion in the use of UAV systems in agriculture is a result of the developing market of drones and sensing solutions attached to them, rendering them economically affordable. In addition, the establishment of formal regulations for UAV operations and the simplification and automatization of the operational and analysis processes had a significant contribution on the increasing popularity of these systems. Data pertaining to the weather conditions of the investigated area were also of great importance as well as soil parameters of the farm at hand. An additional way of getting the data was via in situ manual measurements, involving measurements such as crop height, plant growth, and crop maturity. Finally, data concerning topographic, irrigation, and fertilization aspects were presented with approximately equal frequency.

**Figure 12:**
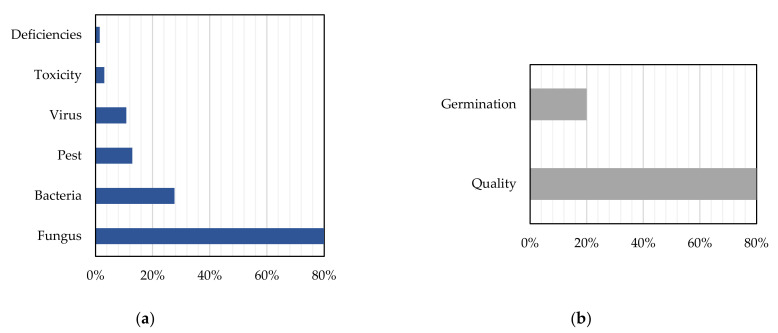
Distribution of the most usual output features of the machine learning algorithms regarding: (**a**) Disease detection and (**b**) Crop quality.

As far as disease detection is concerned, Red-Green-Blue (RGB) images appear to be the most usual input data for the ML algorithms (in 62% of the publications). Normally, deep learning methods like CNNs are implemented with the intention of training a classifier to discriminate images depicting healthy leaves, for example, from infected ones. CNNs use some particular operations to transform the RGB images so that the desired features are enhanced. Subsequently, higher weights are given to the images having the most suitable features. This characteristic constitutes a significant advantage of CNNs as compared to other ML algorithms, when it comes to image classification [79]. The second most common input data came from either multispectral or hyperspectral measurements originated from spectroradiometers, UAVs, and satellites. Concerning the investigated diseases, fungal diseases were the most common ones with diseases from bacteria following, as is illustrated in Figure 12a. This kind of disease can cause major problems in agriculture with detrimental economic consequences [96]. Other examined origins of crop diseases were, in descending order of frequency, pests, viruses, toxicity, and deficiencies.

Images were also the most used input data for weed detection purposes. These images were RGB images that originated mainly from in situ measurements as well as from UGVs and UAVs and, secondarily, multispectral images from the aforementioned sources. Finally, other parameters that were observed, although with lower frequency, were satellite multispectral images, mainly due to the considerably low resolution they provide, video recordings, and hyperspectral and greyscale images. Concerning crop recognition, the majority of the studies used data coming mostly from satellites and, secondarily, from in situ manual measurements. This is attributed to the fact that most of the studies in this category concern crop classification, a sector where satellite imaging is the most widely used data source owing to its potential for analysis of time series of extremely large surfaces of cultivated land. Laboratory measurements followed, while RGB and greyscale images as well as hyperspectral and multispectral measurements from UAVs were observed with lower incidence.

The input data pertaining to crop quality consisted mainly of RGB images, while X-ray images were also utilized (for seed germination monitoring). Additionally, quality parameters, such as color, mass, and flesh firmness, were used. There were also two studies using spectral data either from satellites or spectroradiometers. In general, the studies belonging in this sub-category dealt with either crop quality (80%) or seed germination potential (20%) (Figure 12b). The latter refers to the seed quality assessment that is essential for the seed production industry. Two studies were found about germination that both combined X-ray images analysis and ML.

Concerning soil management, various soil properties were taken into account in 65.7% of the studies. These properties included salinity, organic matter content, and electrical conductivity of soil and soil organic carbon. Usage of weather data was also very common (in 48.6% of the studies), while topographic and data pertaining to the soil moisture content (namely the ratio of the water mass over the dry soil) and crop properties were presented with lower frequency. Additionally, remote sensing, including satellite and UAV multispectral and hyperspectral data, as well as proximal sensing, to a lesser extent, were very frequent choices (in 40% of the studies). Finally, properties associated with soil temperature, land type, land cover, root microbial dynamics, and groundwater salinity make up the rest of data, which are labeled as “other” in the corresponding graph of Figure 11.

In water management, weather data stood for the most common input data (appeared in the 75% of the studies), with ET being used in the vast majority of them. In many cases, accurate estimation of ET (the summation of the transpiration via the plant canopy and the evaporation from plant, soil, and open water surface) is among the most central elements of hydrologic cycle for optimal management of water resources [97]. Data from remote sensors and measurements of soil water content were also broadly used in this category. Soil water availability has a central impact on crops’ root growth by affecting soil aeration and nutrient availability [98]. Stem water potential, appearing in three studies, is actually a measure of water tension within the xylem of the plant, therefore functioning as an indicator of the crop’s water status. Furthermore, in situ measurements, soil, and other parameters related to cumulative water infiltration, soil and water quality, field topography, and crop yield were also used, as can be seen in Figure 11.

Finally, in what concerns livestock management, motion capture sensors, including accelerometers, gyroscopes, and pedometers, were the most common devices giving information about the daily activities of animals. This kind of sensors was used solely in the studies investigating animal welfare. Images, audio, and video recordings came next, however, appearing in both animal welfare and livestock production sub-categories. Physical and growth characteristics followed, with slightly less incidence, by appearing mainly in livestock production sub-category. These characteristics included the animal’s weight, gender, age, metabolites, biometric traits, backfat and muscle thickness, and heat stress. The final characteristic may have detrimental consequences in livestock health and product quality [99], while through the measurement of backfat and muscle thickness, estimations of the carcass lean yield can be made [100].

## 5. Discussion and Main Conclusions

The present systematic review study deals with ML in agriculture, an ever-increasing topic worldwide. To that end, a comprehensive analysis of the present status was conducted concerning the four generic categories that had been identified in the previous review by Liakos et al. [12]. These categories pertain to crop, water, soil, and livestock management. Thus, by reviewing the relative literature of the last three years (2018–2020), several aspects were analyzed on the basis of an integrated approach. In summary, the following main conclusions can be drawn:

- The majority of the journal papers focused on crop management, whereas the other three generic categories contributed almost with equal percentage. Considering the review paper of [12] as a reference study, it can be deduced that the above picture remains, more or less, the same, with the only difference being the decrease of the percentage of the articles regarding livestock from 19% to 12% in favor of those referring to crop management. Nonetheless, this reveals just one side of the coin. Taking into account the tremendous increase in the number of relative papers published within the last three years (in particular, 40 articles were identified in [12] comparing to the 338 of the present literature survey), approximately 400% more publications were found on livestock management. Another important finding was the increasing research interest on crop recognition.
- Several ML algorithms have been developed for the purpose of handling the heterogeneous data coming from agricultural fields. These algorithms can be classified in families of ML models. Similar to [12], the most efficient ML models proved to be ANNs. Nevertheless, in contrast to [12], the interest also been shifted towards EL, which can combine the predictions that originated from more than one model. SVM completes the group with the three most accurate ML models in agriculture, due to some advantages, such as its high performance when it works with image data [101].
- As far as the most investigated crops are concerned, mainly maize and, secondarily, wheat, rice, and soybean were widely studied by using ML. In livestock management, cattle along with sheep and goats stood out constituting almost 85% of the studies. Comparing to [12], more species have been included, while wheat and rice as well as cattle, remain important specimens for ML applications.
- A very important result of the present review study was the demonstration of the input data used in the ML algorithms and the corresponding sensors. RGB images constituted the most common choice, thus, justifying the broad usage of CNNs due to their ability to handle this type of data more efficiently. Moreover, a wide range of parameters pertaining to weather as well as soil, water, and crop quality was used. The most common means of acquiring measurements for ML applications was remote sensing, including imaging from satellites, UAVs and UGVs, while in situ and laboratory measurements were also used. As highlighted above, UAVs are constantly gaining ground against satellites mainly because of their flexibility and ability to provide images with high resolution under any weather conditions. Satellites, on the other hand, can supply time-series over large areas [102]. Finally, animal welfare-related studies used mainly devices such as accelerometers for activity recognition, whereas those ones referring to livestock production utilized primary physical and growth characteristics of the animal.

As can be inferred from the geographical distribution (illustrated in Figure 6) in tandem with the broad spectrum of research fields, ML applications for facilitating various aspects of management in the agricultural sector is an important issue on an international scale. As a matter of fact, its versatile nature favors convergence research. Convergence research is a relatively recently introduced approach that is based on shared knowledge between different research fields and can have a positive impact on the society. This can refer to several aspects, including improvement of the environmental footprint and assuring human’s health. Towards this direction, ML in agriculture has a considerable potential to create value.

**Figure 13:**
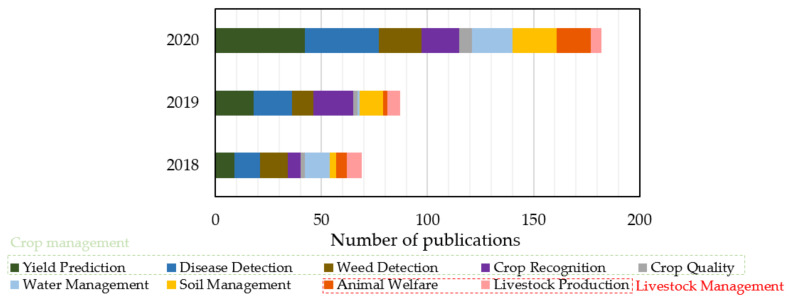
Temporal distribution of the reviewed studies focusing on machine learning in agriculture, which were published within 2018–2020.

Another noteworthy finding of the present analysis is the capturing of the increasing interest on topics concerning ML analyses in agricultural applications. More specifically, as can be shown in Figure 13, an approximately 26% increase was presented in the total number of the relevant studies, if a comparison is made between 2018 and 2019. The next year (i.e., 2020), the corresponding increase jumped to 109% against 2019 findings; thus, resulting in an overall 164% rise comparing with 2018. The accelerating rate of the research interest on ML in agriculture is a consequence of various factors, following the considerable advancements of ICT systems in agriculture. Moreover, there exists a vital need for increasing the efficiency of agricultural practices while reducing the environmental burden. This calls for both reliable measurements and handling of large volumes of data as a means of providing a wide overview of the processes taking place in agriculture. The currently observed technological outbreak has a great potential to strengthen agriculture in the direction of enhancing food security and responding to the rising consumers’ demands.

In a nutshell, ICT in combination with ML, seem to constitute one of our best hopes to meet the emerging challenges. Taking into account the rate of today’s data accumulation along with the advancement of various technologies, farms will certainly need to advance their management practices by adopting Decision Support Systems (DSSs) tailored to the needs of each cultivation system. These DSSs use algorithms, which have the ability to work on a wider set of cases by considering a vast amount of data and parameters that the farmers would be impossible to handle. However, the majority of ICT necessitates upfront costs to be paid, namely the high infrastructure investment costs that frequently prevent farmers from adopting these technologies. This is going to be a pressing issue, mainly in developing economies, where agriculture is an essential economic factor. Nevertheless, having a tangible impact is a long-haul game. A different mentality is required by all stakeholders so as to learn new skills, be aware of the potential profits of handling big data, and assert sufficient funding. Overall, considering the constantly increasing recognition of the value of artificial intelligence in agriculture, ML will definitely become a behind-the-scenes enabler for the establishment of a sustainable and more productive agriculture. It is anticipated that the present systematic effort is going to constitute a beneficial guide to researchers, manufacturers, engineers, ICT system developers, policymakers, and farmers and, consequently, contribute towards a more systematic research on ML in agriculture.

## Figures and Tables

**Table 1:** Summary of the most commonly used evaluation metrics of the reviewed studies.

Name	Formula
Accuracy	(TP + TN)/(TP + FP + FN + TN)
Recall	TP/(TP + FN)
Precision	TP/(TP + FP)
Specificity	TN/(TN + FP)
F1 score	(2 × Recall × Precision)/(Recall + Precision)

**Table 2:** List of the tables appearing in the Appendix A related to: (a) the categories and sub-categories of the machine learning applications in agriculture (Table A1, Table A2, Table A3, Table A4, Table A5, Table A6, Table A7, Table A8 and Table A9) and (b) the abbreviations of machine learning models and algorithms (Table A10 and Table A11, respectively).

Table	Content
A1	Crop Management: Yield Prediction
A2	Crop Management: Disease Detection
A3	Crop Management: Weed Detection
A4	Crop Management: Crop Recognition
A5	Crop Management: Crop Quality
A6	Water Management
A7	Soil Management
A8	Livestock Management: Animal Welfare
A9	Livestock Management: Livestock Production
A10	Abbreviations of machine learning models
A11	Abbreviations of machine learning algorithms

## Appendix A

In this section, the reviewed articles are summarized within the corresponding Tables as described in Table 2.

**Table A1:** Crop Management: Yield Prediction.

Ref	Crop	Input Data	Functionality	Models/Algorithms	Best Output
[103]	Coffee	Weather data, soil fertility	Prediction of Robusta coffee yield by using various soil fertility properties	ELM, RF, MLR	ELM: Model with SOM, K, S: RMSE = 496.35 kgha^−1^, MAE = 326.40 kgha^−1^
[104]	Maize	Weather and satellite spectral data	Silage maize yield estimation via Landsat 8 OLI data	BRT, RFR, SVR, GPR	BRT: R = 0.89, RMSE = 4.66
[105]	Maize	Soil properties, topographic, multispectral aerial images	Prediction of corn yield and soil properties (SOM, CEC, Mg, K, pH)	RF, ANN, SVM, GBM, Cubist	(1) Corn yield: RF (R^2^ = 0.53); (2) SOM: NN (R^2^ = 0.64); (3) CEC: NN (R^2^ = 0.67); (4) K: SVM (R^2^ = 0.21); (5) Mg: SVM (R^2^ = 0.22); (6) pH: GBM (R^2^ = 0.15)
[106]	Cotton	Satellite spectral data	Cotton yield estimation	ANN	(1) 2013: Yield vs. CI (R = −0.2–0.60), best ANN (R = 0.68); (2) 2014: Yield vs. CI (R = −0.79–0.84), best ANN (R = 0.86)
[107]	Apple	RGB images	Detection and estimation of the number of apples in canopy images	MLR	Yield relative error = −10–13%, Yield relative error STD = 28% of average tree yield
[108]	Maize	Crop data—CERES model, satellite spectral data	Forecasting spring maize yield from Landsat-8 images	SVM, RF, DT, LDA, KNN	RS: SVM: Acc = 97%, RMSE = 397 kgha^−1^
[109]	Maize, soybean	Satellite spectral data	Estimation of corn and soybean yield via Landsat and SPOT images	MLR, ANN	R^2^ values: (1) Maize: ANN: 0.92, (2) Soybean: ANN: 0.90
[110]	Turmeric	Soil fertility, weather data	Forecasting oil yield produced from turmeric rhizomes	ANN	Μultilayer-feed-forward NN with 12 nodes: R^2^ = 0.88
[111]	Sunflower	Plant height, SPAD	Prediction of sunflower seed yield	PLSR, ANN	(1) ANN: RMSE = 0.66 tha^−1^, R^2^ = 0.86; (2) PLSR: RMSE = 0.93 tha^−1^, R^2^ = 0.69
[112]	Pistachio	Irrigation, soil characteristics	Estimation of pistachio yield in orchards	MLR, ANN	Acc values: ANN: 90%, MLR: 28%
[113]	Rice	Weather data, irrigation, planting area, fertilization	Evaluation of feature subsets for prediction of paddy crop yield	ANN, SVR, KNN, RF	Forward Feature Selection: RF: RMSE = 0.085, MAE = 0.055, R = 0.93
[114]	Potato	Satellite spectral data	Prediction of potato yield via Sentinel 2 satellite data	MLR, RQL, LB, SVM, RF, MARS, KNN, ANN	(1) Reduced dataset: LB: MAE = 8.95%, R^2^ = 0.89; (2) No feature selection: SVM: MAE = 8.64%, R^2^ = 0.93; (3) 1–2 months prior to harvest: RF: MAE = 8.71%, R^2^ = 0.89
[115]	Wheat	Satellite spectral data	Prediction of wheat yield	SVM, RF, ANN	R^2^ values: (1) SVM: 0.74; (2) RF: 0.68; (3) ANN: 0.68
[116]	Soybean, Maize	Hydrological, weather and satellite spectral data	Prediction of soybean and corn yields	DNN, RF, SVM, MARS, ERT, ANN	DNN (1) Corn: 21–33% more accurate (2) Soybean: 17–22% more accurate
[117]	Wheat, barley	Multispectral images from UAV	Prediction of barley and wheat yields	CNN	(1) Early growth phase(<25%): MAE = 484.3 kgha^−1^, MAPE = 8.8%; (2) Later growth phase(>25%): MAE = 484.3 kgha^−1^, MAPE = 8.8%
[118]	Strawberry	Multispectral images from UAV	Detection and counting of strawberry species for yield prediction	CNN	Faster RCNN: (1) Detection: MaP = 0.83 (at 2 m), MaP = 0.72 (at 3 m); (2) Count: Acc = 84.1%, Average occlusion = 13.5%
[119]	Rice	Weather data, irrigation, planting area, fertilization	Prediction of paddy fields yield	ANN, MLR, SVR, KNN, RF	ANN-MLR: R = 0.99, RMSE = 0.051, MAE = 0.041
[120]	Soybean	Weather and satellite spectral data	Prediction of soybean yield in 15 states of USA	CNN, LSTM	2011–2015: End-of-season RMSE = 329.53 kgha^−1^, R^2^ = 0.78
[121]	Maize	Satellite spectral data	Prediction of maize yield	MLR, RF, SVM	RF: (1) yield: R^2^ = 0.6; (2) GNDVI: R^2^ = 0.48; Best monitoring period: Crop age = 105–135 days
[122]	Mango	Multispectral data from UGV	Estimation of mango maturity level by simulating imaging devices of optical filters	SVM	Estimation of dry matter by using a 4-sensor device with 4 filters: R^2^ = 0.69
[123]	Rapeseed, barley, wheat	EC, STI, gamma radiometrics and weather data	Forecasting crop yield	RF	RMSE = 0.36–0.42 t/ha, Lin’s CCC = 0.89–0.92
[53]	Maize	Genetic information of hybrids, soil and weather data	Prediction of maize yield	DNN	(1) With predicted weather data: RMSE = 12% of average yield, 50% of STD; (2) Using ideal weather data: RMSE = 11% of average yield, 46% of STD
[124]	Rice	RGB leaf images	Prediction of nutrient deficiencies (P, N, K) in image leaves from paddy fields	ANN	Acc = 77%
[125]	Rice	RGB and multispectral images from UAV	Estimation of rice grain yield	CNN	R^2^ values: (1) Only RGB images: 0.424–0.499; (2) RGB and multispectral images: 0.464–0.511
[126]	Maize	Satellite spectral data, crop modeling data	Estimation of end-of-season and early maize yield	RF	(1) Early maize yield: R^2^ = 0.53, RMSE = 271 kgha^−1^, MAE = 202 kgha^−1^; (2) End-of-season maize yield: R^2^ = 0.59, RMSE = 258 kg ha^−1^, MAE = 201 kgha^−1^
[127]	Potato	Soil parameters and tillage treatments	Forecasting of organic potato yield	ANN, MLR	(1) MLR: R^2^ = 0.894, RMSE = 0.431, MAE = 0.327; (2) ANN: R^2^ = 0.95, RMSE = 0.431, MAE = 0.327
[128]	Maize	Simulations data, weather and soil data	Prediction of crop yield based on gridded crop meta-models	RF, XGBoost	(1) XGBoost: (a) growing season climate: R^2^ = 0.91, MAE = 0.74, (b) annual climate: R^2^ = 0.92, MAE = 0.66: (2) RF: (a) growing season climate: R^2^ = 0.94, MAE = 0.71, (b) annual climate: R^2^ = 0.95, MAE = 0.58
[129]	Soybean	Satellite spectral data, precipitation and daytime	Forecasting soybean yield	RF, multivariate OLS, LSTM	(1) DOY 16: OLS: MAE = 0.42 Mgha^−1^; (2) DOY 32: LSTM: MAE = 0.42 Mgha^−1^; (3) DOY 48: LSTM: MAE = 0.25 Mgha^−1^; (4) DOY 64: LSTM: MAE = 0.24 Mgha^−1^
[130]	Potato	Topography, soil EC, soil chemistry and multispectral data from ground based sensors	Potato tuber yield prediction via ground based proximal sensing	LR, KNN, EN, SVR	Best models: (1) SVR: 2017: (a) New Brunswick: RMSE = 5.97 tha^−1^, (b) Prince Edward Island: RMSE = 6.60 tha^−1^; (2) 2018: (a) New Brunswick RMSE = 4.62 tha^−1^, (b) Prince Edward Island: RMSE = 6.17 tha^−1^
[131]	Rice, maize, millet, ragi	Weather data	Prediction of various kharif crops yield	MANN, SVR	Overall RMSE = 79.85%
[132]	Wheat	Soil, weather, and satellite spectral data	Winter wheat prediction from four mid-season timings	RF, GPR, SVM, ANN, KNN, DT, BT	(1) RF: R^2^ = 0.81, RMSE = 910–920 kgha^−1^, MAE = 740 kgha^−1^; (2) GPR: R^2^ = 0.78, RMSE = 920–960 kgha^−1^, MAE = 735–767 kgha^−1^
[133]	Maize	Data derived from various cropping systems	Maize grain yield prediction from CA and conventional cropping systems	LDA, MLR, GNB, KNN, CART, SVM	Best results: LDA: Acc = 0.61, Precision = 0.59, Recall = 0.59, F1-score = 0.59
[134]	Soybean	Multispectral, RGB and thermal images from UAV	Estimation of soybean grain yield	DNN, PLSR, RFR, SVR	DNN: (1) Intermediate-level feature fusion: R^2^ = 0.720, Relative RMSE = 15.9%; (2) input-level feature fusion: R^2^ = 0.691, Relative RMSE = 16.8%
[135]	Soybean, Maize	Weather data and soil data	Soybean and corn yield forecasting	CNN-RNN, RF, LASSO, DNN	CNN-RNN: RMSE values (bushels/acre): (1) Soybean: 2016: 4.15, 2017: 4.32, 2018: 4.91; (2) Maize: 2016: 16.48, 2017: 15.74, 2018: 17.64
[136]	Grape	Multispectral images from UAV	Estimation of vineyard final yield	MLP	(1) Only NDVI: RMSE = 1.2 kg/vine, Relative error = 28.7%; (2) Both NDVI ANF VFC: RMSE = 0.9 kg/vine, Relative error = 21.8%
[137]	Rice	Satellite spectral data	Prediction of rice crop yield	RF, SVM	(1) HD NDVI: RF: RMSE = 11.2%, MAE = 9.1%, SVM: RMSE = 8.7%, MAE = 5.6%; (2) HDM NDVI: RF: RMSE = 11.3%, MAE = 9.2%, SVM: RMSE = 8.7%, MAE = 5.6%
[138]	Maize	Fertilization, planting density, soil EC, satellite spectral data	Prediction of corn yield response to nitrogen and seed rate management	CNN	Average value for 9 fields in the USA: RMSE = 0.7
[139]	Sugarcane	Monthly precipitation data	Forecasting of sugarcane yield	RNN	RMSE = 0.31 tha^−1^, MAE = 0.39 tha^−1^, MAPE = 5.18%
[140]	Wheat	Satellite spectral and weather data	Estimation of wheat yield	SVR, RF, Cubist, XGBoost, MLP, GPR, KNN, MARS	SVR: RMSE = 0.55 tha^−1^, R^2^ = 0.77
[141]	Maize, Soybean	Satellite spectral data	Forecasting of maize and soybean yield	MLR, ANN	ANN: (1) Corn: RMSE = 4.83–8.41, R = 0.91–0.99; (2) Soybean: RMSE = 5.18–7.77, R = 0.79–0.99
[142]	Maize	Satellite spectral and weather data	Prediction of maize yield under severe weather conditions	DNN	(1) Drought cases: R = 0.954; (2) Heatwave cases: R = 0.887–0.914
[143]	Rice	Weather data	Paddy yield prediction	ANN	R = 0.78–1.00, MSE = 0.040–0.204
[144]	Maize	Plant population, soil and weather data	Maize yield forecasting in 3 US states of Corn Belt	XGBoost, RF, LASSO, GBM, WEL	WEL: RMSE = 1.138 kgha^−1^
[145]	Maize	Satellite spectral and weather data	Estimation of maize yield	DLS	R^2^ = 0.76, RMSE = 0.038 tha^−1^
[146]	Various crops	Satellite spectral and weather data	Prediction of autumn crops yield	SVR, RF, DNN	RMSE values (×10^4^ tons) SVR = 501.98; RF = 477.45; DNN = 253.74
[147]	Wheat	Multispectral images from UAV	Growth monitoring and yield prediction of wheat in key growth stages	LR, SMLR, PLSR, ANN, RF	Best results: RF: R^2^ = 0.78, RMSE = 0.103
[148]	Cotton	Topographic, weather, soil and satellite spectral data	Within-field yield prediction	RF, GB	Best results: RF: RMSE = 0.20 tha^−1^, CCC = 0.50–0.66
[149]	Cotton	Satellite spectral data	Yield prediction	RF, CART	RF: RMSE = 62.77 Kg ha^−1^, MAPE = 0.32
[150]	Rice	Multispectral images from UAV	Prediction of rice grain yield	RF	RMSE = 62.77 Kg ha^−1^, MAPE = 0.32
[151]	Soybean	Multispectral images from UAV	Yield estimation in soybean	MLP	R = 0.92
[152]	Potato	Weather, irrigation, and satellite spectral data	Forecasting of yield in potato fields at municipal level	RF, SVM, GLM	(1) winter cycle: R^2^ = 0.757, %RMSE = 18.9; (2) summer cycle; R^2^ = 0.858, %RMSE = 14.9
[153]	Sugarcane	Satellite spectral data	Prediction of sugarcane yield	MLR	R^2^ = 0.92–0.99
[154]	Cotton	Multispectral images from UAV	Estimation of cotton yield	ANN, SVR, RFR	ANN: R^2^ = 0.9
[155]	Rice	Weather and soil data	Prediction of rice yields from Blockchain nodes	RF, MLR, GBR, DTR	RF: R^2^ = 0.941, %RMSE = 0.62, MAE = 0.72
[156]	Maize	Multispectral images from UAV	Prediction of maize yield at specific phenological stages	GB	Stage V10: R^2^ = 0.90; Stage VT: R^2^ = 0.93
[157]	Wheat	Satellite spectral and weather data, soil hydraulic properties	Forecasting of wheat yield	RF, MLR	RF: 1 month before harvest: R = 0.85, RMSE = 0.70 tha^−1^, ROC = 0.90
[158]	Maize	Soil and weather data	Estimation of maize yield with publicly available data	LSTM, LASSO, RF, SVR, AdaBoost	LSTM: MAE = 0.83 (buac^−1^), MAPE = 0.48%
[159]	Rice	Soil and weather data	Finding optimal features gathering for forecasting paddy yield	RF, DT, GBM	RF: MSE = 0.07, R^2^ = 0.67;
[160]	Alfalfa	Hyperspectral data from UAV	In-season alfalfa yield forecast	Combination of RF, SVR, KNN	R^2^ = 0.874
[161]	Maize	Multispectral images from UAV	Yield prediction of maize	BPNN, SVM, RF, ELM	SVM: RMSE = 1.099, MAE = 0.886
[162]	Mentha	Satellite spectral data, field inventory data (soil, plant height, biomass)	Mentha crop biomass forecasting	MLP	R^2^ = 0.762, RMSE = 2.74 th^−1^
[163]	Wheat	Multispectral images from UAV	Prediction of wheat grain yield	LR, RF, SVM, ANN	LR: RMSE = 972 kgha^−1^, R^2^ = 0.62
[164]	Maize	Multispectral images from UAV	Prediction of maize yield	RF, RF+R, RF+BAG, SVM, LR, KNN, ANN	RF: R = 0.78, MAE = 853.11 kgha^−1^
[165]	Potato	Hyperspectral data from UAV	Yield prediction at two growth stages	RF, PLSR	RF: R^2^ = 0.63, MAE = 853.11 kgha^−1^
[166]	Carrot	Satellite spectral data	Carrot yield Mapping	RF	R^2^ = 0.82, RMSE = 2.64 Mgha^−1^; MAE = 1.74 Mgha^−1^
[167]	Soybean	multispectral images from UAV	Predicting yield	DT	RMSE = 196 kgha^−1^
[168]	Wheat	Satellite spectral, soil and weather data	Winter wheat yield prediction at a regional level	Combination of LSTM and CNN	R^2^ = 0.75, RMSE = 732 kgha^−1^;
[169]	Potato	Hyperspectral data from UAV	Yield prediction at two growth stages	RF, PLSR	R^2^ values: RF: 0.63; PLSR: 0.81
[170]	Wheat	Satellite spectral and weather data	Winter yield prediction in the Conterminous United States	OLS, LASSO, SVM, RF, AdaBoost, DNN	AdaBoost: R^2^ = 0.86, RMSE = 0.51 tha^−1^, MAE = 0.39 tha^−1^

Acc: Accuracy: CA: Conservation Agriculture; CI: Crop Indices; CEC: Cation Exchange Capacity; CCC: Concordance Correlation Coefficient; DOY: Day Of Year; EC: Electrical Conductivity; HD: Heading Date; HDM: Heading Date to Maturity; K: Potassium; Mg: Magnesium; N: Nitrogen; OLI: Operational Land Imager; P: Phosphorus; RGB: Red-Green-Blue; S: Sulphur; SOM: Soil Organic Matter; SPAD: Soil and Plant Analyzer Development; STI: Soil Texture Information; STD: Standard Deviation; UAV: Unmanned Aerial Vehicle; UGV: Unmanned Ground Vehicle.

**Table A2:** Crop Management: Disease Detection.

Ref	Crop	Input Data	Functionality	Models/Algorithms	Best Output
[171]	Various crops	RGB images	Detection and diagnosis of plant diseases	CNN	Acc = 99.53%
[172]	Melon	Fluorescence, thermal images	Detection of Dickeya dadantii in melon plants	LR, SVM, ANN	ANN: Whole leaves: Acc = 96%; F1 score = 0.99
[173]	Tomato	RGB images	Recognition of 10 plant diseases and pests in tomato plants	CNN	Recognition rate = 96%
[174]	Avocando	Hyperspectral images	Detection of nitrogen and iron deficiencies and laurel wilt disease in avocando	DT, MLP	MLP: Detection at early stage: Acc = 100%
[175]	Maize	RGB images	Examination of nine factors affecting disease detection in maize fields	CNN	Acc values: (1) Original dataset: 76%; Background removed: 79%; (2) Subdivided (full): 87%; (3) Subdivided (reduced): 81%
[176]	Milk thistle	Spectral measurements form spectroradiometer	Identification of Microbotryum silybum in milk thistle plants	MLP-ARD	Acc = 90.32%
[177]	Tomato	Spectral measurements form spectroradiometer	Detection of leaf diseases (target, bacterial spots and late blight) in tomato	KNN	Acc values: (1) Healthy leaves: 100%, (2) Asymptomatic: 100%, (3) Early stage: 97.8%, (4) Late stage: 100%
[178]	Maize	RGB images	Identification of eight types of leaf diseases in maize	CNN	(1) GoogLeNet: Acc = 98.9%; (2) Cifar10: Acc = 98.8%
[179]	Various crops	RGB images	Identification of six plant leaf diseases	RBFN	(1) Early blight: Acc = 0.8914; (2) Common rusts: Acc = 0.8871
[180]	Citrus	RGB images	Detection and classification of citrus diseases	SVM	Acc values: 1st dataset: 97%; 1st and 2nd dataset: 89%; 3rd dataset: 90.4%
[181]	Grape	Multispectral images from UAV	Identification of infected areas	CNN	(1) Color space YUV: Acc = 95.84%; (2) Color space YUV and ExGR: Acc = 95.92%
[182]	Soybeean	RGB images	Detection and classification of three leaf diseases in soybeans	SVM	(1) Healthy: Acc = 82%; (2) Downy mildew: Acc = 79%; (3) Frog eye: Acc = 95.9%; (4) Septoria leaf blight: Acc = 90%
[183]	Millet	RGB images	Identification of fungal disease (mildew) in pearl millet	CNN	Acc = 95.00%, Precision = 90.50%, Recall = 94.50%, F1 score = 91.75%
[184]	Maize	RGB images from UAV	Detection of northern leaf blight in maize	CNN	Acc = 95.1%
[185]	Wheat	RGB images from UAV	Classification of helminthosporium leaf blotch in wheat	CNN	Acc = 91.43%,
[186]	Avocado	RGB images, multispectral images	Detection of laurel wilt disease in healthy and stressed avocado plants in early stage	MLP, KNN	Healthy vs. Nitrogen deficiency using 6 bands images: (1) MLP: Acc = 98%; (2) KNN: Acc = 86%
[187]	Basil	RGB images	Identification and classification of five types of leave diseases in four kinds of basil leaves	DT, RF, SVM, AdaBoost, GLM, ANN, NB, KNN, LDA	RF: Acc = 98.4%
[188]	Various crops	RGB images	Identification of several diseases on leaves	CNN	Acc values: (1) Healthy: 89%; (2) Mildly diseased: 31%; (3) Moderately diseased: 87%; (4) Severely diseased: 94%
[189]	Tea	RGB images from UAV	Identification of tea red Scab, tea leaf blight and tea red leaf spot diseases in tea leaves	SVM, DT, RF, CNN	CNN: Acc values: (1) tea red Scab: 0.7; (2) tea leaf blight: 1.0; (3)tea red leaf spot: 1.0
[190]	Wheat	Hyperspectral images from UAV	Detection of yellow rust in wheat plots	CNN	Acc = 0.85
[191]	Grape	RGB images	Detection of grapevine yellows in red grapes	CNN	Sensitivity = 98.96% Specificity = 99.40%
[192]	Maize	RGB images from UAV	Detection of northern leaf blight in maize	CNN	Acc = 0.9979, F1 score = 0.7153
[193]	Sugar beet	RGB images	Detection and classification of diseased leaf spots in sugar beet	CNN	Acc = 95.48%
[194]	Various crops	RGB images	Identification of various plant leaf diseases	CNN	Acc = 96.46%
[195]	Strawberry	RGB images	Detection of powdery mildew in strawberry leaves	LDA	(1) Artificial lighting conditions: recall = 95.26%, precision = 95.45%, F1 score = 95.37%; (2) Natural lighting conditions: recall = 81.54%, precision = 72%, F1 score = 75.95%
[196]	Various different crops	RGB images	Detection of diseased plants	DL	Acc = 93.67%
[197]	Citrus	Hyperspectral images from UAV	Detection of canker disease on leaves and immature fruits	RBFN, KNN	RBFN: Acc values: (a) asymptomatic: 94%, (b) early stage: 96%, (c) late stage: 100%
[198]	Grape	RGB images	Detection of diseased vine on leaves	SVM	Acc = 95%
[199]	Wheat	RGB images	Identification of three leaf diseases in wheat	CNN	Acc values: (1) Septoria: 100%; (2) Tan Spot: 99.32%; (3) Rust: 99.29%
[200]	Grape	Spectral measurements form spectroradiometer	Classification of Flavescence dorée disease in grapevines	SVM, LDA	SVM: Acc = 96%
[201]	Papaya	RGB images	Recognition of five papaya diseases	SVM	Acc = 90%, Precision = 85.6%
[202]	Rice	RGB images	Recognition and classification of rice infected leaves	KNN, ANN	ANN: Acc = 90%, Recall = 88%
[203]	Tomato	Hyperspectral images from UAV	Detection of bacterial spot and target spot on tomato leaves	MLP, STDA	MLP: Acc values: (a) bacterial spot: 98%, (b) target spot: 97%
[204]	Squash	Hyperspectral images from UAV and laboratory measurements	Classification of powdery mildew in squash	RBFN	Acc values: (1) Laboratory: Asymptomatic: 82%, Late stage: 99%; (2) Field conditions: Early stage: 89%, Late disease stage: 96%
[205]	Tomato	Hyperspectral images from UAV and laboratory measurements	Detection of bacterial spot and target spot on tomato leaves	RBFN, STDA	Field conditions: Acc values: (a) Healthy vs. BS: 98%, (b) Healthy vs. TS: 96%, (c) Healthy vs. TYLC: 100%
[206]	Tomato	RGB images	Identification of various diseases in tomato	CNN	Acc values: (1) PV dataset: 98.4%; (2) 2nd dataset: 98.7%; (3) Field data: 86.27%
[79]	Walnut	RGB images	Identification of anthracnose infected leaves	CNN	Acc values: (1) RGB: 95.97%; (2) Grayscale: 92.47%; (3) Fast Fourier: 92.94%
[207]	Various crops	RGB images	Classification of infected leaves	DBN	Acc = 0.877, Sensitivity = 0.862, Specificity = 0.877
[208]	Grape	Multispectral images from UAV	Detection of Mildew disease in vineyards	CNN	Acc values: (1) Grapevine-level: 92%; (2) Leaf level: 87%
[209]	Rice	RGB images, videos	Video detection of brown spot, stem borer and sheath blight in rice	CNN	(1) Brown spot: Recall = 75.0%, Precision = 90.0%; (2) Stem borer: Recall = 45.5%, Precision = 71.4%; (3) Sheath blight: Recall = 74.1%, Precision = 90.9%
[210]	Cassava	RGB images	Detection and classification of diseased leaves of fine-grain cassava	CNN	Acc = 93%
[211]	Banana	Satellite spectral data, Multispectral images from UAV, RGB images from UAV	Detection of banana diseases in different African landscapes	RF, SVM	RF: Acc = 97%, omissions error = 10%; commission error = 10%; Kappa coefficient = 0.96
[212]	Tomato	RGB images	Detection of early blight, leaf mold and late blight on tomato leaves	CNN	Acc = 98%
[213]	Pepper	Spectral reflectance at 350–2500 nm	Detection of fusarium disease in pepper leaves	ANN, NB, KNN	ΚNN: Average success rate = 100%
[214]	Tomato	Spectral measurements form spectroradiometer	Detection of fusarium disease on pepper leaves	CNN	Acc = 98.6%
[215]	Citrus	Multispectral images from UAV	Detection of citrus greening in citrus orchards	SVM, KNN, MLR, NB, AdaBoost, ANN	AdaBoost: Acc = 100%
[216]	Soybean	RGB images	Prediction of charcoal rot disease in soybean	GBT	Sensitivity = 96.25%, specificity = 97.33%
[217]	Wheat	RGB images from UAV	Detection of wheat lodging	RF, CNN, SVM	CNN: Acc = 93%
[218]	Tomato	Weather data	Prediction of powdery mildew disease in tomato plants	ELM	Acc = 89.19%, AUC = 88.57%
[219]	Soybean	RGB images	Diagnosis of soybean leaf diseases	CNN	Acc = 98.14%
[220]	Potato	RGB images	Identification of early and late blight disease	NB, KNN, SVM	SVM: Average Acc = 99.67%
[221]	Various crops	RGB images	Quantification of uncertainty in detection of plant diseases	BDL	Mean softmax probability values: (1) Healthy: 0.68; (2) Non-Healthy: 0.72;
[222]	Coffee	Satellite spectral data	Identification of coffee berry necrosis via satellite imagery	MLP, RF, NB	NB: Acc = 0.534
[223]	Tomato	RGB images	Recognition of blight, powdery mildew, leaf mold fungus and tobacco mosaic virus diseases	CNN	Faster RCNN: mAP = 97.01%
[224]	Maize	RGB images	Diagnosis of northern leaf blight, gray leaf spot, and common rust diseases	CNN	Acc = 98.2%; macro average precision = 0.98
[225]	Grape	RGB images	Detection of black measles, black rot, leaf blight and mites on leaves	CNN	mAP = 81.1%
[226]	Grape	Weather data, expert input (disease incidence form visual inspection)	Forecasting downy mildew in vineyards	GLM, LASSO, RF, GB	GB: AUC = 0.85
[227]	Maize	RGB images	Detection of northern leaf blight in maize	CNN	mAP = 91.83%
[228]	Onion	RGB images	Detection of downy mildew symptoms in onions field images	WSL	mAP@0.5 = 74.1–87.2%
[229]	Coffee	RGB images	Detection of coffee leaf rust via remote sensing and wireless sensor networks	CNN	F1 score = 0.775, *p*-value = 0.231
[230]	Tomato	Weather data, multispectral images captured from UAV	Detection of late blight disease	CNN	Acc values: AlexNet: (1) Transfer learning: 89.69%; (2) Feature extraction: 93.4%,
[231]	Rice	RGB images	Detection of brown rice planthopper	CNN	Average recall rate = 81.92%, average Acc = 94.64%
[232]	Grape	UAV multispectral images, depth map information	Detection of vine diseases	CNN	VddNet: Accuracy = 93.72%
[233]	Apple	RGB images	Identification of apple leaf diseases (S, FS, CR)	CNN	Improved VGG16: Acc = 99.40%(H), 98.04% (S), 98.33%(FS), 100%(CR)
[234]	Cotton	UAV multispectral images	Disease classification of cotton root rot	KM, SVM	KM: Acc = 88.39%, Kappa = 0.7198

Acc: Accuracy; AUC: Area Under Curve; CR: Cedar Rust; ExGR: Excess Green Minus Excess Red; FS: Frogeye Spot; H: Healthy; mAP: mean Average Precision; RGB: Red-Green-Blue; S: Scab; TYLC: Tomato Yellow Leaf Curl; UAV: Unmanned Aerial Vehicle; VddNet: Vine Disease Detection Network.

**Table A3:** Crop Management: Weed Detection.

Ref	Input Data	Functionality	Models/Algorithms	Best Output
[235]	RGB images	Classification of thinleaf (monocots), broa leaf (dicots) weeds	AdaBoost with NB	Acc values: (1) Original dataset: 98.40%; (2) expanded dataset: 94.72%
[236]	RGB images from UAV	Detection of weeds in bean, spinach fields	CNN	Acc values: (1) Bean field: 88.73%; (2) Spinach field: 94.34%
[237]	RGB images	Detection of four weed species in sugar beet fields	SVN, ANN	Overall Acc: SVM: 95.00%; Weed classification: SVM: 93.33%; Sugar beet plants: SVM: 96.67%
[238]	RGB images from UAV, multispectral images	Detection of Gramineae weed in rice fields	ANN	Best system: 80% < M/M_GT_ < 108%, 70% < MP < 85%
[239]	RGB images	Classification of crops (three species) and weeds (nine species)	CNN	Average Acc: 98.21±0.55%
[240]	Multispectral and RGB images from UAV	Weed mapping between and within crop rows, (1) cotton; (2) sunflower	RF	Weed detection Acc: (1) Cotton: 84% (2) Sunflower: 87.9%
[241]	Hyperspectral images	Recognition of three weed species in maize crops	RF	Mean correct classification rate: (1) Zea mays: 1.0; (2) Convolvulus arvensis: 0.789; Rumex: 0.691; Cirsium arvense 0.752
[242]	RGB images from UAV	Detection of weeds in early season maize fields	RF	Overall Acc = 0.945, Kappa = 0.912
[243]	RGB images from UAV	Weed mapping and prescription map generation in rice field	FCN	Overall Acc = 0.9196, mean intersection over union (mean IU) = 0.8473
[244]	Handheld multispectral data	Weed detection in maize and sugar beet row-crops with: (1) spectral method; (2) spatial; (3) both methods	SVM	Mean detection rate: (1) spectral method: 75%; (2) spatial: 79%; (3) both methods: 89%
[245]	Multispectral images from UAV	Development of Weed/crop segmentation, mapping framework in sugar beet fields	DNN	AUC: (1) background: 0.839; (2) crop: 0.681; (3) weed: 0.576
[246]	RGB images	Classification of potato plant and three weed species	ANN	Acc = 98.1%
[247]	RGB images	Estimation of weed growth stage (18 species)	CNN	Maximum Acc = 78% (Polygonum spp.), minimum Acc = 46% (blackgrass), average Acc = 70% (the number of leaves) and 96% for deviation of two leaves
[248]	Multispectral images	Classification of corn (crop) and silver beet (weed)	SVM	Precision = 98%, Acc = 98%
[249]	RGB images	Classification of Carolina Geranium within strawberry plants	CNN	F1 score values: (1) DetectNet: (0.94, highest); (2) VGGNet: 0.77; (3) GoogLeNet: 0.62
[250]	RGB images	Classification of weeds in organic carrot production	CNN	Plant-based evaluation: Acc = 94.6%, Precision = 93.20%, Recall = 97.5%, F1 Score = 95.32%
[251]	Grayscale images from UGV	Recognition of Broad-leaved dock in grasslands	CNN, SVM	VGG-F: Acc = 96.8%
[252]	Multispectral images from UAV	Mapping of Black-grass weed in winter wheat fields	CNN	Baseline model: AUC = 0.78; Weighted kappa = 0.59; Average misclasssification rate = 17.8%
[253]	RGB images	Segmentation of rice and weed images at seedling stage in paddy fields	FCN	Semantic segmentation: Average Acc rate = 92.7%
[254]	RGB images from UGV	Creation of multiclass dataset for classification of eight Australian rangelands weed species	CNN	RS-50: Average Acc = 95.7%, average inference time = 53.4 ms per image
[255]	RGB images	Evaluation of weed detection, spraying and mapping system. Two Scenarios: (1) artificial weeds, plants; (2) real weeds, plants	CNN	Scenario: (1) Acc = 91%, Recall = 91%; (2) Acc = 71%, Precision = 78% (for plant detection and spraying Acc)
[256]	RGB images	Detection of goldenrod weed in wild blueberry crops	LC, QC	QC: Acc = 93.80%
[257]	RGB images	Detection of five weed species in turfgrass	CNN	Precision values: Dollar weed: VGGNet (0.97); old world diamond-flower: VGGNet (0.99); Florida pusley: VGGNet (0.98); annual bluegrass: DetectNet (1.00)
[258]	RGB images	Detection of three weed species in perennial ryegrass	CNN	Precision values: Dandelion: DetectNet (0.99); ground ivy: VGGNet (0.99), spotted spurge: AlexNet (0.87)
[259]	RGB images, multispectral images from UGV	Crop-weed classification along with stem detection	FCN	Overall: Mean precision = 91.3%, Mean recall = 96.3%
[260]	RGB images	Identification of crops (cotton, tomato) and weeds (velvetleaf and nightsade)	CNN, SVM, XGBoost, LR	Densenet and SVM: micro F1 score = 99.29%
[261]	Videos recordings	Classification of two weeds species in rice field	ANN, KNN	Acc values: Right channel (76.62%), Left channel (85.59%)
[262]	RGB images	Weed and crop discrimination in paddy fields	MCS, SRF, SVM	Acc values: Right channel (76.62%), Left channel (85.59%)
[263]	Gray-scale and RGB images	Weed and crop discrimination in carrot fields	RF	Acc = 94%
[264]	Multispectral and RGB images	Discrimination of weed and crops with similar morphologies	CNN	Acc = 98.6%
[265]	RGB images	Detection of C. sepium weed and sugar beet plants	CNN	mAP = 0.751–0.829 APs@IoU0.5 = 0.761–0.897
[266]	RGB images	Recognition of eight types of weeds in rangelands	CNN, RNN	DeepWeeds dataset: Acc = 98.1%
[267]	Multispectral images from UAV	Weed estimation on lettuce crops	SVM, CNN	F1 score values: (1) SVM: 88%; (2) CNN-YOLOv3: 94%; (3) Mask R-CNN: 94%
[268]	RGB images	Examination of pre-trained DNN for improvements in weed identification	CNN	(1) Xception: improvement = 0.51%; (2) Inception-Resnet: improvement = 1.89%
[269]	RGB images from UAV	Detection of five weeds in soybean fields	CNN	Faster RCNN: precision = 065, recall = 0.68, F1 score = 0.66, IoU = 0.85
[270]	RGB images	Detection of goose grass weed in tomato, strawberry fields	CNN	(1) Strawberry: (a) entire plant: F1 score = 0.75, (b) leaf blade: F1 score = 0.85; (2) Tomato: (a) entire plant: F1 score = 0.56, (b) leaf blade: F1 score = 0.65
[271]	Video recordings	Detection of five weed species in Marfona potato fields	ANN	Correct classification rate = 98.33%
[272]	In situ measurements, satellite spectral data	Identification of gamba grass in pasture fields	XGBoost	Balanced Acc = 86.9%
[273]	RGB images from UAV, satellite spectral data	Weed maps creation in oat fields	RF	Acc values: (1) Subset A: 89.0%; (2) Subset B: 87.1%
[274]	In situ measurements, RGB images from UAV	Identification of Italian ryegrass in early growth wheat	DNN	Presicion = 95.44%, recall = 95.48%, F score = 95.56%
[275]	RGB images from UGV	Weed detection evaluation of a spraying robot in potato fields on: (1) Image-level; (2) application-level; (3) field-level	CNN	YOLOv3: (1) Image-level: recall = 57%, precision = 84%; (2) application-level: plants detected = 83%; (3) field-level: correct spraying = 96%
[276]	RGB images from UGV	Detection of four weed species in maize and bean crops	CNN	Average precision = 0.15–0.73
[277]	RGB images from UAV	Detection of Colchicum autumnale in grassland sites	CNN	U-Net: Precision = 0.692, Recall = 0.886, F2 score = 0.839
[278]	RGB images from UAV	Weed mapping of Rumex obtusifolius in native grasslands	CNN	VGG16: Acc = 92.1%, F1 score = 78.7%

Acc: Accuracy; AUC: Area under Curve; IoU: Intersection over Union; mAP: mean Average Precision; RGB: Red-Green-Blue; UAV: Unmanned Aerial Vehicle; UGV: Unmanned Ground Vehicle.

**Table A4:** Crop Management: Crop Recognition.

Ref	Crop	Input Data	Functionality	Models/Algorithms	Best Output
[279]	Various crops	Satellite spectral data	Classification of early-season crops	RF	Beginning of growth stage: acc = 97.1%, kappa = 93.5%
[280]	Various crops	Satellite spectral and phenological data	Identification of various crops from remote sensing imagery	SVM, RF, DF	DF: (1) 2015: overall acc = 88%; (2) 2016: overall acc = 85%
[281]	Maize, Rice, Soybean	Satellite spectral data	Three-dimensional classification of various crops	CNN, SVM, KNN	CNN: (1) 2015: overall acc = 0.939, kappa = 0.902; (2) 2016: overall acc = 0.959, kappa = 0.924
[282]	Various crops	Satellite spectral data, in situ data	Identification of crops growing under plastic covered greenhouses	DT	Overall acc = 75.87%, Kappa = 0.63
[283]	Various crops	Satellite data, phenological, in situ data	Classification of various crops	NB, DT, KM	KM: overall acc = 92.04%, Kappa = 0.7998
[284]	Cabbage, Potato	RGB images from UAV, in situ data	Classification of potato and cabbage crops	SVM, RF	SVM: overall acc = 90.85%
[285]	Various crops	Satellite spectral data	Classification of various crops	SVM	Overall acc = 94.32%
[286]	Various crops	Satellite spectral data, in situ data	Classification of various crops in large areas	EBT, DT, WNN	EBT: overall acc = 87%
[287]	Various crops	Satellite spectral data, in situ data	Classification of various crops	SVM	overall acc = 92.64%
[288]	Various crops	Field location, in situ and satellite spectral data	Classification of six crops with small sample sizes	FFNN, ELM, MKL, SVM	MKL: accuracy = 92.1%
[289]	Wolfberry, Maize, Vegetables	Satellite spectral data	Crop classification in cloudy and rainy areas	RNN	Landsat-8: overall acc = 88.3%, Kappa = 0.86
[290]	Maize, Canola, Wheat	Satellite spectral data, in situ data	Crop classification	RF, ANN, SVM	RF: overall acc = 0.93, Kappa = 0.91
[291]	Various crops	Satellite spectral data	Classification of various crop types	Combination of FCN-LSTM	Acc = 86%, IoU = 0.64
[292]	Various crops	Satellite spectral data	Crop classification of various crops	LightGBM	Highest acc: 92.07%
[293]	Maize, Peanut, Soybeans, Rice	Satellite spectral and in situ data	Prediction of different crop types	FCN, SVM, RF	Best crop mapping: FCN: acc = 85%, Kappa = 0.82
[294]	Various crops	Satellite spectral and in situ data	Classification of early growth crops	CNN, RNN, RF	Highest Kappa: 1D CNN: 0.942
[295]	Various crops	Satellite spectral and in situ data	Classification of various crops	CNN, LSTM, RF, XGBoost, SVM	CNN: acc = 85.54%, F1 score = 0.73
[296]	Various crops	Satellite spectral data	Classification of parcel-based crops	LSTM, DCN	DCN: overall acc = 89.41%
[297]	Various crops	Satellite spectral data	Classification of crops in farmland parcel maps	LSTM, RF, SVM	LSTM: overall acc = 83.67%, kappa = 80.91%
[298]	Various crops	Satellite spectral data, in situ data	Crop classification	SVM, RF, CNN-RNN, GBM	Pixel R-CNN: acc = 96.5%
[299]	Zea mays, Canola, radish	Grayscale testbed data	Classification of the crops	SVM	Quadratic SVM: Precision = 91.87%, Recall = 91.85%, F1 score = 91.83%
[300]	Rice	Morphological data	Classification of two rice species (Osmancik-97 and Cammeo)	LR, MLP, SVM, DT, RF, NB, KNN	LR: acc = 93.02%
[301]	Soybean	Hyperspectral data, seed properties	Discrimination of 10 soybean seed varieties	TS-FFNN, SIMCA, PLS-DA, BPNN	TS-FFNN in terms of identification Acc, stability and computational cost
[302]	Cotton	Hyperspectral data, seed properties	Identification of seven cotton seed varieties: (1) Full spectra, (2) Effective wavelengths	PLS-DA, LGR, SVM, CNN	(1) Full spectra: CNN-SoftMax: 88.838%; (2) Effective wavelengths: CNN-SVM: 84.260%
[303]	Various plants	RGB images of leaves	Recognition of 15 plant species of Swedish leaf dataset	CNN	Macro average: (1) Precision = 0.97, (2) Recall = 0.97, (3) F1 score = 0.97
[304]	Various shrubs and trees	RGB images of leaves	Identification of 30 shrub and trees species	RF, SVM, AdaBoost, ANN	SVM: acc = 96.5–98.4%
[305]	Various plants	RGB images of leaves	Identification of seven plant species	BPNN, SOM, KNN, SVM	BPNN: Recognition rate = 92.47%
[306]	Various crops	Satellite spectral data	Crop classification	SVM	SVM (RBF): overall acc values: (1) 2016: 88.3%; (2) 2017: 91%; (3) 2018: 85.00%
[307]	Various crops	Satellite spectral data	Crop classification	FCN	3D FCN: overall acc = 97.56%, Kappa = 95.85%
[308]	Cotton, Rice, Wheat, Gram	Satellite spectral data	Crop classification	RF, KM	RF: acc = 95.06%
[309]	Various crops	Satellite spectral data	Crop classification	SVM, RF, CART	RF: overall acc = 97.85%, Kappa = 0.95
[310]	Various crops	Satellite spectral data, in situ data	Crop classification	RF	overall acc = 75%, Kappa = 72%
[311]	Maize, Soybean	Satellite spectral data	Crop classification	RF, MLP, LSTM	LSTM: confidence interval = 95%
[312]	Various crops	Satellite spectral and in situ data	Crop classification	XGBoost, SVM, RF, MLP, CNN, RNN	CNN: overall acc = 96.65%
[313]	Rice	Satellite spectral data	Crop classification	CNN, SVM, RF, XGboost, MLP	CNN: overall acc = 93.14%, F1 score = 0.8552
[314]	Various crops	Satellite spectral and in situ data	Crop classification	RF	Overall acc = 0.94, Kappa = 0.93
[315]	Various crops	Satellite spectral data	Crop classification	CNN, LSTM, SVM	CNN: overall acc = 95.44%, Kappa = 94.51%
[316]	Various crops	Satellite spectral data	Crop classification prior to harvesting	DT, KNN, RF, SVM	RF: overall acc = 81.5%, Kappa = 0.75
[317]	Various crops	Satellite spectral data	Crop classification	CNN	Overall acc = 98.19%
[318]	Various crops	Satellite spectral data	Crop classification	SVM, DA, DT, NNL	NNL: F1 score = 0.88
[319]	Banana, Rice, Sugarcane, Cotton	Satellite spectral and in situ data	Crop classification	SVM	Overall acc = 89%
[320]	Various crops	Satellite spectral and in situ data	Crop classification	RF	Overall acc = 93.1%

Acc: Accuracy; IoU: Intersection over Union; RGB: Red-Green-Blue; UAV: Unmanned Aerial Vehicle.

**Table A5:** Crop Management: Crop Quality.

Ref	Crop	Input Data	Functionality	Models/Algorithms	Best Output
[64]	Apples	Quality features, (flesh firmness, soluble solids, fruit mass and skin color)	Classification of apple total quality: very poor, poor, medium, good and excellent	FIS, ANFIS	FIS: acc values: (1) 2005: 83.54%; 2006: 92.73%; 2007: 96.36%
[321]	Pepper	RGB images, quality features (color, mass and density of peppers)	Recognition of pepper seed quality	BLR, MLP	MLP: 15 traits, stability = 99.4%, predicted germination = 79.1%, predicted selection rate = 90.0%
[322]	Soybeans	Satellite spectral and soil data	Estimation of crop gross primary productivity	RF, ANN	ANN: R^2^ = 0.92, RMSE = 1.38 gCdm^−2^
[323]	Wheat	RGB images captured by UAV	Estimation of aboveground nitrogen content combining various VI and WFs	PLSR, PSO-SVR	PSO-SVR: R^2^ = 0.9025, RMSE = 0.3287
[324]	Millet, rye, maize	RGB images captured in laboratory	Assessment of grain crops seed quality	CNN	Faster R-CNN: (1) Pearl millet: mAP = 94.3%; (2) rye: mAP = 94.2%, (3) Maize: mAP = 97.9%
[325]	Jatropha curcas	X-ray imaging	Prediction of vigor and germination	LDA	Acc values: Fast germination: 82.08%; Slow germination: 76.00%; Non-germinated: 88.24%
[326]	Various legumes	Spectral data form spectroradiomener	Estimation of five warm-season legumes forage quality	PLS, SVM, GP	SVM: All five crops: $\mathrm{Acc}=\frac{R_{cv}^{2}}{R_{v}^{2}}$ = 0.92–0.99, IVTD: $\mathrm{Acc}=\frac{R_{cv}^{2}}{R_{v}^{2}}$ = 0.42–0.98
[327]	Forage grass	X-ray imaging	Prediction of vigor and seed germination	LDA, PLS-DA, RF, NB, SVM	PLS-DA: Acc values: (1) Vigor: FT-NIR: 0.61, X-ray: 0.68, Combination: 0.58; (2) Germination: FT-NIR: 0.82, X-ray: 0.86, Combination: 0.82
[328]	Tomato	RGB images	Dimensions and mass estimation for quality inspection	(1) DSM, (2) Dimensions (CNN), (3) Mass estimation on: (a) MMD (BET, GPR, SVR, ANN, GPR), (b) EDG (BET, GPR, SVR, ANN)	(1) DSM: precision = 99.7%; MAE values: (2) Width (2.38), Length (2.58); (3) Mass estimation: (a) MMD (4.71), (b) EDG (13.04)
[329]	Peach	Hyperspectral images	Estimation of soluble solids content	SAE-RF	R^2^ = 0.9184, RMSE = 0.6693

Acc: Accuracy; DSM: Detection and Segmentation Module; EDG: Estimated Dimensions Geometry; IVTD: In Vitro True Digestibility; RGB; Red-Green-Blue; MMD: Manually Measured Dimensions; mAP: mean Average Precision; PSO: Particle Swarm Optimization; RGB; Red-Green-Blue; SAE: Stacked AutoEncoder; VI: Vegetation Indices; WF: Wavelet Features.

**Table A6:** Water management.

Ref	Property	Input Data	Functionality	Models/Algorithms	Best Output
[330]	Crop water status	Weather data, crop water status, thermal images	Prediction of vineyard’s water status. Scenario A: with RT; Scenario B: without RT	REPTree	(1) Scenario A: prediction: R^2^ = 0.58, RMSE = 0.204 MPa; (2) Scenario B: prediction: R^2^ = 0.65, RMSE = 0.184 MPa.
[331]	Crop water status	Crop water status, hyperspectral data	Discrimination of stressed and non-stressed vines	RF, XGBoost	RF: Acc = 83.3%, Kappa = 0.67
[332]	Groundwater level	Water table depth, weather data	Prediction of water table depth	LSTM, FFNN,	LSTM: R^2^ = 0.789–0.952
[333]	Irrigation scheduling	Weather, irrigation, soil moisture, yield data	Prediction of weekly irrigation plan in jojoba orchards	DTR, RFR, GBRT, MLR, BTC	(1) Regression: GBRT: Acc = 93%; (2) Classification: GBRT: Acc = 95%
[334]	Crop water status	Water status, multispectral UAV data	Estimation of vineyard water status	MLR, ANN	ANN: R^2^ = 0.83
[335]	ET	Weather data	Estimation of daily ET_o_	ELM, WANN	ELM: RMSE values: Region case A: 0.1785 mm/day; Region case B: 0.359 mm/day
[336]	ET	Weather data	Estimation of daily ET_o_	RF, M5Tree, GBDT, XGBoost, SVM, RF	XGBoost: RMSE = 0.185–0.817 mmday^−1^
[337]	Soil water content	Weather data, volumetric soil moisture content	Prediction of one-day-ahead volumetric soil moisture content	FFNN, LSTM	LSTM: R^2^ > 0.94
[338]	Infiltration	Field data, moisture content, cumulative infiltration of soil	Estimation of cumulative infiltration of soil	SVM, ANN, ANFIS	ANFIS: RMSE = 0.8165 cm, CC = 0.9943
[339]	Soil water content	Weather data, soil moisture difference, ultraviolet radiation	Prediction of soil moisture	SVR	R = 0.98, R^2^ = 0.96, MSE = 0.10
[340]	Soil water content	Simulated soil moisture data, weather data	Forecasting of monthly soil moisture for: Scenario A: upper; Scenario B: lower layers	ELM	(1) Scenario A: RRMSE = 19.16%; (2) Scenario B: RRMSE = 18.99%
[341]	ET	Weather and in situ crop data	Estimation of actual ET Scenario A: rainfed maize field under non-mulching; Scenario B: partial plastic film mulching	ANN, SVM	ANN: Scenario A: ET = 399.3 mm, RMSE = 0.469, MAE = 0.376; Scenario B: ET = 361.2 mm, RMSE = 0.421, MAE = 0.322
[342]	Infiltration and infiltration rate	Soil and hydraulic data	Prediction of cumulative infiltration and infiltration rate in arid areas	ANFIS, SVM, RF	SVM: RMSE values: cumulative infiltration: 0.2791 cm, infiltration rate: 0.0633 cmh^−1^
[343]	Water quality	NIR spectroscopy.	Estimation of water pollution level	CNN	RMSE = 25.47 mgL^−1^
[344]	ET	Weather data, simulated ET data	Estimation of ET_o_: (1) 2011–2015; (2) 2016–2017	LSTM	(1) Predictions in 3 sites: R^2^ > 0.90; (2) All sites: RMSE = 0.38–0.58 mmday^−1^
[345]	Soil water content	Weather data, potential ET, simulated soil moisture data	Estimation of soil moisture	FFNN, Ross-IES	FFNN: RMSE = 0.15–0.25, NSE = 0.71–0.91
[346]	ET	Weather data, simulated ET data, soil data	Estimation of daily kikuyu grass crop ET	RT, SVR, MLP, KNN, LGR, MLR, BN, RFC	RFC: R = 0.9936, RMSE = 0.183 mmday^−1^, MRE = 6.52%
[347]	Drought	Weather data	Evaluation of farmers’ draught perception	RF, DT	Most influential parameters: farmer’s age, education level, years of experience and number of cultivated land plots
[348]	ET	Weather and soil data; simulated ET	Prediction of daily potato ET	ANN, AdaBoost, KNN	KNN: R^2^ = 0.8965, RMSE = 0.355 mm day^−1^, MSE = 0.126 mm day^−1^
[349]	Soil water erosion	In situ data, geological, and weather data	Susceptibility mapping of soil erosion from water	RF, GP, NB	RF: Acc = 0.91, kappa = 0.94, POD = 0.94
[350]	ET, drought	Weather data, simulated ET index	Prediction of drought	SVR	Fuzzy-SVR: R^2^ = 0.903, RMSE = 0.137, MAE = 0.105
[351]	ET	Weather data, simulated ET_o_	Estimation of daily ET_o_	CNN, ANN, XGBoost, RF	CNN: (1) Regional: R^2^ = 0.91, RMSE = 0.47; (2) Local: R^2^ = 0.92, RMSE = 0.37
[352]	ET	Weather data	Estimation of daily ET_o_	ELM, ANN, RF	ELM: R^2^ = 0.920, MAE = 0.394 mmday^−1^
[353]	ET	Weather data	Prediction of ET in semi-arid and arid regions	CART, CCNN, SVM	SVM: (1) Station I: R^2^ = 0.92; (1) Station II: R^2^ = 0.97
[354]	Pan evaporation	Weather data	Prediction of monthly pan evaporation	ELM, ANN, M5Tree	ELM: R^2^ = 0.864–0.924, RMSE = 0.3069–0.4212
[355]	ET	Weather data, simulated ET_o_	Evaluation of ML algorithms in daily reference ET prediction	Cubist, SVM, ANN, MLR	Cubist: R^2^ = 0.99, RMSE = 0.10 mmday^−1^, MAE = 0.07 mmday^−1^
[356]	ET	Weather data, simulated ET	Estimation of ET_o_	SVM, MLP, CNN, GRNN, GMDH	SVM: R = 0.96–1.00, ME = 95–99%
[357]	Drought	Weather data, simulated Palmer Z-index values	Estimation of Palmer drought severity index	ANN, DT, LR, SVM	ANN: R = 0.98, MSE = 0.40, RMSE = 0.56
[358]	Water quality	In-situ water quality data, hyperspectral, satellite data.	Estimation of inland water quality.	LSTM, PLSR, SVR, DNN	DNN: R^2^ = 0.81, MSE = 0.29, RMSE = 0.54
[359]	Groundwater	In-situ water quality data, hyperspectral, satellite spectral data	Estimation of water quality	DT	Acc = 81.49%, ROC = 87.75%
[360]	Groundwater	Weather data, ET, satellite spectral data, land use	Estimation of groundwater withdrawals	RF	R^2^ = 0.93, MAE = 4.31 mm, RMSE = 13.50 mm
[361]	Groundwater nitrate concentration	Various geo-environmental data	Comparison of different ML models for estimating nitrate concentration	SVM, Cubist, RF, Bayesian-ANN	RF: R^2^ = 0.89, RMSE = 4.24, NSE = 0.87

Acc: Accuracy; CC: Coefficient of Correlation; ET: Evapotranspiration; ET_o_: reference EvapoTranspiration; ROC: Receiver Operating Characteristic; ME: Model Efficiency; NSE: Nash-Sutcliffe model efficiency Coefficient; POD: Probability Of Detection.

**Table A7:** Soil management.

Ref	Property	Input Data	Functionality	Models/Algorithms	Best Output
[362]	Soil organic matter	Soil properties, spectrometer NIR data	Estimation of soil organic matter	ELM, SVM	TRI-ELM: R^2^ = 0.83, RPIQ = 3.49
[363]	Soil microbial dynamics	Microbial dynamics measurements from root samples	Prediction of microbial dynamics: (1) BP; (2) PS and (3) ACCA	ANN, SVR, FIS	SCFIS: (1) BP: RMSE = 1350000, R^2^ = 1.00; (2) PS: RMSE = 45.28, R^2^ = 1.00; (3) ACCA: RMSE = 271, R^2^ = 0.52
[364]	Soil salinity	Soil salinity, hyperspectral data, satellite data	Prediction of soil salinity	Bootstrap BPNN	BPNN with hyperspectral data: R^2^ = 0.95, RMSE = 4.38 g/kg
[365]	Soil properties	Simulated topographic attributes, satellite data	Prediction of SOC, CCE, clay content	Cu, RF, RT, MLR	(1) CCE: Cu: R^2^ = 0.30, RMSE = 9.52; (2) SOC: Cu, RF: R^2^ = 0.55; (3) Clay contents: RF: R^2^ = 0.15, RMSE = 7.86
[366]	Soil organic matter	Soil properties, weather data, terrain, satellite spectral data	Prediction of soil organic matter	DT, BDT, RF, GBRT	GBRT: ME = 1.26 g/kg, RMSE = 5.41 g/kg, CCC = 0.72
[367]	Soil organic matter	soil properties, satellite, land cover, topographic, weather data	Prediction of soil organic matter	CNN, RF, XGBoost	XGBoost: ME = 0.3663 g/kg, MSE = 1.0996 g/kg
[368]	Electrical conductivity	soil properties, simulated electrical conductivity	Prediction of soil electrical conductivity	MLP	MLP: WI = 0.780, E_NS_ = 0.725, E_LM_ = 0.552
[369]	Soil moisture content	Hyperspectral images data, UAV, soil moisture content data samples	Estimation of soil moisture content	RF, ELM	RF: R^2^ = 0.907,RMSEP = 1.477, RPD = 3.396
[370]	Soil temperature	Weather data	Estimation of soil temperature at various depths	ELM, GRNN, BPNN, RF	ELM: RMSE = 2.26–2.95 °C, MAE = 1.76–2.26 °C, NSE = 0.856–0.930, CC = 0.925–0.965
[371]	SOC	Soil properties, vis-NIR spectral data	Estimation of SOC	RF	R^2^ = 0.74–0.84, RMSEP = 0.14–0.18%, RPD = 1.98–2.5
[372]	Soil properties	Soil properties, visible-NIR, MIR spectral data	Prediction of total carbon, cation exchange capacity and SOC	PLSR, Cu, CNN	CNN: R^2^ = 0.95–0.98
[373]	Soil properties	Soil properties, simulated organic, mineral samples, soil spectral data	Estimation of various soil properties	CNN	RMSE values: OC: 28.83 g/kg, CEC: 8.68 cmol^+^/kg, Clay: 7.47%, Sand: 18.03%, pH: 0.5 g/kg, N: 1.52 g/kg
[374]	Soil moisture content, soil ET	Soil properties, water, weather and crop data	Estimation of soil moisture content and soil ET	NN-RBF	Soil MC: RMSE = 0.428, RSE = 0.985, MSE = 0.183, RPD = 8.251
[375]	Soil salinity	Soil salinity, crop field temperature	Estimation of leaching water requirements for saline soils	Naive Bayes classifier	Acc = 85%
[376]	Soil erosion	Weather data, satellite, soil chemical data	Estimation of soil erosion susceptibility	Combination of GWR-ANN	GWR-ANN: AUC = 91.64%
[377]	Soil fertility	Spectral, weather data, EC, soil properties	Prediction of soil fertility and productivity	PLS	(1) Productivity: RMSEC = 0.20 T/ha, RMSECV = 0.54 T/ha, R^2^ = 0.9189; (2) Organic matter: R^2^ = 0.9345, RMSECV = 0.54%; (3) Clay: R^2^ = 0.9239, RMSECV = 5.28%
[378]	Soil moisture	Multispectral images from UAV, in situ soil moisture, weather data.	Retrieval of surface soil moisture	BRT, RF, SVR, RVR	BRT: MAE = 3.8%
[379]	Soil moisture	Soil samples, simulated PWP, field capacity data	Estimation of PWP and field capacity	ANN, KNN, DL	R^2^ = 0.829, R = 0.911, MAE = 0.027
[380]	Soil temperature	Weather data	Estimation of soil temperature	GMDH, ELM, ANN, CART, MLR	ELM: R = 0.99
[381]	Soil moisture	Soil samples, on-field thermal, simulated soil moisture data	Estimation of soil moisture content	ANN, SVM, ANFIS	SVM: R = 0.849, RMSE = 0.0131
[382]	Gully erosion	Geological, environmental, geographical data	Evaluation of gully erosion susceptibility mapping	RF, CDTree, BFTree, KLR	RF: AUC = 0.893
[383]	Groundwater salinity	Topographic, groundwater salinity data	Evaluation of groundwater salinity susceptibility maps	StoGB, RotFor, BGLM	BGLM: Kappa = 0.85
[384]	Heavy metals transfer	Soil and crop properties	Identification of factors related to heavy metals transfer	RF, GBM, GLM	RF: R^2^ = 0.17–0.84
[385]	Land suitability	Soil properties, weather, topography data	Prediction of land suitability maps	SVM, RF	RF: Kappa = 0.77, overall acc = 0.79
[386]	SOC	Soil properties, satellite, simulated environmental data	Prediction of SOC	MLR, SVM, Cu, RF, ANN	RF: R^2^ = 0.68
[387]	Electrical conductivity, SOC	Soil properties, weather data	Electrical conductivity and SOC prediction	GLM	(1) EC: MSPE = 0.686, MAPE = 0.635; (2) OC: MSPE = 0.413, MAPE = 0.474
[388]	SOC, soil moisture	Proximal spectral data, electrical conductivity, soil samples data	Prediction of SOC and soil moisture 3D maps	Cu, RF	Cu: R^2^ = 0.76, CCC = 0.84, RMSE = 0.38%
[389]	Soil aggregate stability	Soil samples data	Prediction of soil aggregate stability	GLM, ANN	ANN: R^2^ = 0.82
[390]	SOC	Soil samples, weather, topographic, satellite data	Prediction of SOC	Cu, RF, SVM, XGBoost, KNN	Best SOC prediction: RF: RMSE = 0.35%, R^2^ = 0.6
[391]	Soil moisture	In situ soil moisture, satellite data	Estimation of surface soil moisture	SVM, RF, ANN, EN	RF: NSE = 0.73
[392]	SOC	Composite surface soil, satellite, weather data	Prediction of SOC	SVM, ANN, RT, RF, XGBoost, DNN	DNN: MAE = 0.59%, RMSE = 0.75%, R^2^ = 0.65, CCC = 0.83
[393]	Gully erosion	Topographic, weather, soil data	Mapping of gully erosion susceptibility	LMT, NBTree, ADTree	LMT: AUC = 0.944
[394]	Gully erosion	Satellite spectral data	Identification of gully erosion	LDA, SVM, RF	Best overall acc: RF: 98.7%
[395]	Gully erosion	Satellite, weather, land type maps data	Gully erosion mapping	LGR	Acc = 68%, Kappa = 0.42

ACCA: Aminoyclopropane-1-carboxylate; AUC: Area Under Curve; BP: Bacterial Population; CC: Coefficient of Correlation; CCC: Concordance Correlation Coefficient; CCE: Calcium Carbonate Equivalent; ET: EvaporoTransporation; MIR: Mid InfraRed; NSE: Nash-Sutcliffe model efficiency Coefficient; NIR: Near-InfraRed; PS: Phosphate Solubilization; PWP: Permanent Wilting Point; RPIQ: Ratio of Performance to Interquartile Range; RPD: Relative Percent Deviation; SOC: Soil Organic Carbon; WI: Willmott’s Index.

**Table A8:** Livestock Management: Animal Welfare.

Ref	Animal	Input Data	Functionality	Models/Algorithms	Best Output
[396]	Swine	3D, 2D video images	Detection of pigs tail posture as a sign of tail biting	LMM	Low vs. not low tails: Acc = 73.9%, Sensitivity = 88.4%, Specificity = 66.8%
[397]	Sheep	Accelerometer and gyroscope attached to the ear and collar of sheep	Classification of Grazing and Rumination Behavior in Sheep	RF, SVM, KNN, Adaboost	RF: Highest overall acc: collar: 92%; ear: 91%
[398]	Sheep	Accelerometer, gyroscope data	Classification of sheep behavior (lying, standing and walking)	RF	Acc = 95%, F1-score = 91–97% for: ear: 32 Hz, 7 s, collar: 32 Hz, 5 s
[399]	Swine	RGB images	Recognition of pigs feeding behavior	CNN	Faster R-CNN: Precision = 99.6%, recall = 86.93%
[400]	Swine	RGB images, depth images	Recognition of lactating sow postures	CNN	Faster R-CNN: Sow posture: (1) Recumbency: night: 92.9%, daytime: 84.1%; (2) Standing: at night: 0.4%, daytime: 10.5% (3) Sitting: night: 0.55%, daytime: 3.4%
[401]	Cattle, Sheep, sheepdog	Audio field recordings data	Classification of animals’ vocalization	SVM	Acc: cattle: 95.78%, sheep: 99.29%, dogs: 99.67%
[402]	Cattle	Accelerometer data	Detection of sheep rumination.	SVM	Acc = 86.1%
[403]	Sheep	Ear-borne accelerometer data, observation recordings	Classification of grazed sheep behavior Scenario A: walking, standing, lying, grazing Scenario B: active/inactive Scenario C: body posture	CART, SVM, LDA, QDA	(1) Scenario A: SVMAcc: 76.9%; (2) Scenario B: CART Acc: 98.1%; (3) Scenario C: Acc: LDA 90.6%
[404]	Goat	On-farm videos, weather data	Classification of goats behavior (1) Anomaly detection (2) Feeding/non-feeding	KNN, SVR, CNN	(1) Most accurate: KNN: Acc = 95.02–96.5%; (2) Faster R-CNN: Eating: 55.91–61.33 %, Non-feeding (Resting): 79.91–81.53 %
[405]	Cattle, sheep	UAV Video data	Counting and classification of cattle, sheep	CNN	Mask R-CNN: Cattle: Acc = 96%; Sheep: Acc = 92%
[406]	Cattle	Accelerometer data	Prediction of dairy cows behavior at pasture	XGBoost, SVM, AdaBoost, RF	Best predictions for most behaviours: XGBoost: sensitivity = 0.78
[407]	Cattle	Pedometers	Detection of early lameness in dairy cattle	RF, KNN	RF: acc = 91%
[408]	Cattle	Environmental heat stressors data	Evaluation of heat stressors influence in dairy cows physiological responses	RF, GBM, ANN, PLR	RF: (1) RR: RMSE = 9.695 respmin^−1^; (2) ST: RMSE = 0.334 °C
[409]	Cattle	Diets nutrient levels data	Prediction of dairy cows diet energy digestion	ELM, LR, ANN, SVM	Best performance: kernel-ELM: (1) DE: R^2^ = 08879, MAE = 4.0606; (2) ED: R^2^ = 0899, MAE = 2.3272
[410]	Cattle	Routine herd data	Detection of lameness in dairy herds	GLM, RF, GBM, XGBoost, CART	GBM: AUC = 0.75, Sensitivity = 0.58, Specificity = 0.83
[411]	Poultry	Air quality data	Early prediction of Coccidiosis in poultry farms	KNN	AUC = 0.897–0.967
[412]	Cattle	On-farm questionnaires, clinical and milk records	Prediction of mastitis infection in dairy herds	RF	CONT vs. ENV: Acc = 95%, PPV = 100%, NPV = 95%
[413]	Cattle	Location (transceiver) and accelerometer data	Detection of dairy cows in estrus	KNN, LDA, CART, BPNN, KNN	BPNN: specificity = 85.71%
[414]	Cattle	Mid-NIR spectral data using spectrometer	Prediction of bovine tuberculosis in dairy cows	CNN	Accuracy = 71%, sensitivity = 0.79, specificity = 0.65
[415]	Cattle	Metabolomics data from serum samples	Evaluation of metabotypes existence in overconditioned dairy cows	RF, NB, SMO, ADT	ADT: acc = 84.2%
[416]	Cattle	Accelerometer data	Classification of cows’ behavior	GBDT, SVM, RF, KNN	GBDT: acc = 86.3%, sensitivity = 80.6%
[417]	Sheep	Gyroscope and accelerometer ear sensors	Detection of lame and non-lame sheep in three activities	RF, SVM, MLP, AdaBoost	RF: overall acc = 80%
[418]	Cattle	Activity and rumination data	Prediction of calving day in cattle	RNN, RF, LDA, KNN, SVM	RNN/LSTM: Sensitivity = 0.72, Specificity = 0.98

AUC: Area Under Curve; Cont: Contagious; DE: Digestible Energy; ED: Energy Digestibility; ENV: Environmental; DWT: Discrete Wavelet Transform; MFCCs: Mel-Frequency Cepstral Coefficients; NIR: Near InfraRed; NPV: Negative Predictive Value; PTZ: Pan-Tilt-Zoom; PPV: Positive Predictive Value; RGB: Red-Green-Blue; RR: Respiration Rate; ST: Skin Temperature.

**Table A9:** Livestock Management: Livestock Production.

Ref	Animal	Input Data	Functionality	Models/Algorithms	Best Output
[419]	Cattle	Depth images in situ BCS evaluation data	Estimation of BCS, Scenario A: HER = 0.25; Scenario B: HER = 0.5	CNN	Scenario A: Acc = 78%; Scenario B: Acc = 94%
[420]	Swine	Weather, physiological data	Prediction of piglets temperature Scenario A: skin-surface; Scenario B: hair-coat; Scenario C: core	DNN, GBR, RF, GLR	Best prediction: Scenario C: DNN: error = 0.36%
[421]	Poultry	Depth, RGB images data	Classification of flock of chickens’ behavior	CNN	Acc = 99.17%
[422]	Cattle	Accelerometer, observations recordings data	Classification of cattle behaviour Scenario A: grazing; Scenario B: standing; Scenario C: ruminating	RF	Highest F-scores: RF: Scenario A: 0.914; Scenario B: 0.89; Scenario C: 0.932
[423]	Sheep	Phenotypic, weather data	Prediction of on-farm water and electricity consumption on pasture based Irish dairy farms	BAG, ANN, MT	Scenario 3: MT: R = 0.95, MAE = 0.88 μm, RMSE = 1.19
[424]	Cattle	Milk production, environmental data	Prediction of on-farm water and electricity consumption on pasture based Irish dairy farms	CART, RF, ANN, SVM	Electricity consumption prediction: SVM: relative prediction error = 12%
[425]	Goat	RGB data	Detection of dairy goats from surveillance video	CNN	Faster R-CNN: Acc = 92.49 %
[426]	Cattle	Animal feed, machinery, milk yield data	Estimation of energy use targets for buffalo farms	ANN	30.5 % of total energy input can be saved if targeted inputs are followed
[427]	Cattle	3D images data	Prediction of liveweight and carcass characteristics	ANN, SLR	ANN: Liveweight: R^2^ = 0.7, RMSE = 42; CCW: R^2^ = 0.88, RMSE = 14; SMY: R^2^ = 0.72, RMSE = 14
[428]	Swine	RGB images	Detection and pig counting on farms	CNN	MAE = 1.67, RMSE = 2.13, detection speed = 42 ms per image
[429]	Sheep	Biometric traits, body condition score data	Prediction of commercial meat cuts and carcass traits	MLR, ANN, SVR, BN	SVM: Neck weight: R^2^ = 0.63, RMSE = 0.09 kg; HCW: R^2^ = 0.84, RMSE = 0.64
[430]	Cattle	Data produced by REIMS	Prediction of beef attributes (muscle tenderness, production background, breed type and quality grade)	SVM, RF, KNN, LDA, PDA, XGBoost, LogitBoost, PLS-DA	Best Acc: SVM: 99%
[431]	Sheep	Carcass, live weight and environmental records	Estimation of sheep carcass traits (IMF, HCW, CTLEAN, GRFAT, LW)	DL, GBT, KNN, MT, RF	Highest prediction of all traits: RF: (1) IMF: R = 0.56, MAE = 0.74; (2) HCW: R = 0.88, MAE = 1.19; (3) CTLEAN: R = 0.88, MAE = 0.76
[432]	Swine	ADG, breed, MT, gender and BBFT	Identification of pigs’ limb condition	RF, KNN, ANN, SVM, NB, GLM, Boost, LDA	RF: Acc = 0.8846, Kappa = 0.7693
[433]	Cattle	Activity, weather data	Prediction of cows protein and fat content, milk yield and actual concentrate feed intake, Scenario (1) only cows with similar heat tolerance; Scenario (2) all cows	ANN	(1) Scenario A: n = 116, 456; R = 0.87; slope = 0.76; (2) Scenario B: n = 665, 836; R = 0.86; slope = 0.74
[434]	Cattle	Animal behavior, feed intake, estrus events data	Detection of estrus in dairy heifers	GLM, ANN, RF	RF: Acc = 76.3–96.5%
[435]	Cattle	Infrared thermal images	Estimation of deep body temperature	LRM, QRM	Higher correlation: QRM: R^2^ = 0.922
[436]	Cattle	Liveweight, biophysical measurements data	Prediction of Carcass traits and marbling score in beef cattle	LR, MLP, MT, RF, SVM	SVM: carcass weight: R = 0.945, MAE = 0.139; EMA: R = 0.676, MAE = 4.793; MS: R = 0.631, MAE = 1.11

ACFW: Adult Clean Fleece Weight; ADG: Average Daily Gain; AFD: Adult Fibre Diameter; AGFW: Adult Greasy Fleece Weight; ASL: Adult Staple Length; ASS: Adult Staple Strength; BBFT: Bacon/BackFat Thickness; BCS: Body Condition Score; CCW: Cold Carcass Weights; CTLEAN: Computed Tomography Lean Meat Yield; DBT: Deep Body Temperature; EMA: Eye Muscle Area; GWAS: Genome-Wide Association Studies; GRFAT: Greville Rule Fat Depth; HER: Human Error Range; IMF: IntraMuscular Fat; HCW: Hot Carcass Weight; LW: Loin Weight; MS: Marbling Score; MT: Muscle Thickness; REIMS: Rapid Evaporative Ionization Mass Spectrometry; RGB: Red-Green-Blue; SMY: Saleable Meat Yield.

**Table A10:** Abbreviations for machine learning models.

Abbreviation	Model
ANN	Artificial Neural Network
BM	Bayesian Models
DL	Deep Learning
DR	Dimensionality Reduction
DT	Decision Trees
EL	Ensemble Learning
IBM	Instance Based Models
SVM	Support Vector Machine

**Table A11:** Abbreviations for machine learning algorithms.

Abbreviation	Model	Model
AdaBoost	EL	Adaptive Boosting
ADT	DT	Alternating Decision Trees
ANFIS	ANN	Adaptive-Neuro Fuzzy Inference Systems
ARD	BM	Automatic Relevance Determination
Bayesian-ANN	ANN	Bayesian Artificial Neural Network
BAG	EL	Bagging Algorithm
BDT	DT	Bagging Decision Trees
BDL	BM,ANN	Bayesian Deep Learning
BET	EL	Bagged Ensemble Tree
BGLM	BM, Regression	Bayesian Generalized Linear Model
BLR	Regression	Binary Logistic Regression
BN	BM	Bayesian Network
BPNN	ANN	Back-Propagation Neural Networks
BRT	DT,EL	Boosted Regression Trees
BTC	EL	Boosted Trees Classifiers
CART	DT	Classification And Regression Trees
CCNN	ANN	Cascade Correlation Neural Networks
CDTree	DT	Credal Decision Trees
CNN	ANN	Convolutional Neural Networks
Cu	Regression	Cubist
DBN	ANN	Deep Belief Networks
DF	EL,SVM	Decision Fusion
DLS	Regression	Damped Least Squares
DNN	ANN	Deep Neural Networks
DTR	DT, Regression	Decision Tree Regression
EBT	DT,EL	Ensemble Bagged Trees
ERT	DT	Extremely Randomized Trees
ELM	ANN	Extreme Learning Machines
EN	Regression	Elastic Net
FCN	ANN	Fully Convolutional Networks
FIS	ANN	Fuzzy Inference System
FFNN	ANN	Feed Forward Neural Networks
GBM	EL	Gradient Boosting Model
GBT	DT	Gradient Tree Boosting
GBR	Regression	Gradient Boosted Regression
GBRT	DT, Regression	Gradient Boosted Regression Trees
GBDT	DT,EL	Gradient Boosted Decision Trees
GLM	Regression	General Linear Model
GMDH	DR	Group Method of Data Handling
GNB	BM	Gaussian Naive Bayes
GP	ΒΜ	Gaussian Processes
GPR	ΒΜ	Gaussian Process Regression
GRNN	ANN	Generalized Regression Neural Networks
GWR	Regression	Geographically Weighted Regression
KM	IBM	K-Means
KNN	IBM	K-Nearest Neighbors
LASSO	Regression	Least Absolute Shrinkage and Selection Operator
LDA	DR	Linear Discriminant Analysis
LightGBM	EL	Light Gradient Boosting Machine
LMT	Regression, DT	Logistic Model Trees
LGR	Regression	LoGistic Regression
LMM	Regression	Linear Mixed Model
LR	Regression	Linear Regression
LSTM	ANN	Long-Short Term Memory
LogitBoost	EL	Logistic Boosting
M5Tree	DT	M5 model Trees
MANN	ANN	Modular Artificial Neural Networks
MARS	Regression	Multivariate Adaptive Regression Splines
MCS	EL	Multiple Classifier System
MKL	DR	Multiple Kernel Learning
MLP	ANN	Multi-Layer Perceptron
MLR	Regression	Multiple Linear Regression
MT	DT	Model Trees
NB	BM	Naïve Bayes
NBTree	BM, DT	Naïve Bayes Trees
NNL	IBM	Nearest Neighbor Learner
OLS	Regression	Ordinary Least Squares
PLSR	Regression	Partial Least Squares Regression
PLS-DA	Regression, DR	Partial Least Squares Discriminant Analysis
QC	Regression	Quadratic Classifier
QDA	DR	Quadratic Discriminant Analysis
QRM	Regression	Quadratic Regression Model
RBFN	ANN	Radial Basis Function Networks
REPTree	DT	Reduced Error Pruning Tree
RFC	EL	Randomizable Filtered Classifier
RFR	EL, Regression	Random Forest Regression
RNN	ANN	Recurrent Neural Network
RQL	Regression	Regression Quantile LASSO
RF	EL	Random Forest
Ross-IES	EL	Ross Iterative Ensemble Smoother
RotFor	EL	Rotation Forest
RVMR	Regression	Relevance Vector Machine Regression
SCFIS	ANN	Subtractive Clustering Fuzzy Inference System
STDA	DR	Stepwise Discriminant Analysis
SMO	SVM	Sequential Minimal Optimization
SMLR	Regression	Stepwise Multiple Linear Regression
SOM	DR	Self-Organising Maps
StoGB	EL	Stochastic Gradient Boosting
SVR	SVM	Support Vector Regression
TS-FNN	ANN	Takagi-Sugeno Fuzzy Neural Networks
XGBoost	EL	Extreme Gradient Boosting
WANN	ANN	Wavelet Artificial Neural Networks
WEL	EL	Weighted Ensemble Learning
WNN	IBM	Weighted Nearest Neighbors
WSL	EL	Weakly Supervised Learning
